# Genomic Dissection of an Enteroaggregative *Escherichia coli* Strain Isolated from Bacteremia Reveals Insights into Its Hybrid Pathogenic Potential

**DOI:** 10.3390/ijms25179238

**Published:** 2024-08-26

**Authors:** Alejandra M. G. Del Carpio, Claudia A. Freire, Fernanda B. Andrade, Roxane M. F. Piazza, Rosa M. Silva, Eneas Carvalho, Waldir P. Elias

**Affiliations:** 1 Laboratório de Bacteriologia, Instituto Butantan, São Paulo 05503-900, Brazil; aledani_7@usp.br (A.M.G.D.C.); claudia.freire.esib@esib.butantan.gov.br (C.A.F.); fernanda.andrade.esib@esib.butantan.gov.br (F.B.A.); roxane.piazza@butantan.gov.br (R.M.F.P.); 2 Departamento de Microbiologia, Imunologia e Parasitologia, Escola Paulista de Medicina, Universidade Federal de São Paulo, São Paulo 04023-062, Brazil; rosa.unifesp@gmail.com

**Keywords:** enteroaggregative *Escherichia coli*, EAEC, bacteremia, hybrid *Escherichia coli*, genomics

## Abstract

*Escherichia coli* is a frequent pathogen isolated from bloodstream infections. This study aimed to characterize the genetic features of EC092, an *E. coli* strain isolated from bacteremia that harbors enteroaggregative *E. coli* (EAEC) genetic markers, indicating its hybrid pathogenic potential. Whole-genome sequencing showed that EC092 belongs to phylogroup B1, ST278, and serotype O165:H4. Genes encoding virulence factors such as fimbriae, toxins, iron-uptake systems, autotransporter proteins (Pet, Pic, Sat, and SepA), and secretion systems were detected, as well as EAEC virulence genes (*aggR*, *aatA*, *aaiC*, and *aap*). EC092 was found to be closely related to the other EAEC prototype strains and highly similar in terms of virulence to three EAEC strains isolated from diarrhea. The genomic neighborhood of *pet*, *pic*, *sat*, *sepA*, and the EAEC virulence genes of EC092 and its three genetically related fecal EAEC strains showed an identical genomic organization and nucleotide sequences. Also, EC092 produced and secreted Pet, Pic, Sat, and SepA in the culture supernatant and resisted the bactericidal activity of normal human serum. Our results demonstrate that the strain EC092, isolated from bacteremia, is a hybrid pathogenic extraintestinal *E. coli* (ExPEC)/EAEC with virulence features that could mediate both extraintestinal and intestinal infections.

## 1. Introduction

Bloodstream infections (BSIs) remain a significant global concern due to their association with high mortality rates [1]. Among the predominant causative agents, *Escherichia coli* is one of the most frequently isolated Gram-negative bacteria [2,3,4,5]. Despite its common presence in the intestinal microbiota of humans and warm-blooded animals [6,7,8], certain pathogenic strains can cause intestinal and/or extraintestinal diseases through the acquisition of virulence genes via horizontal transfer mechanisms [9,10,11]. Extraintestinal pathogenic *E. coli* (ExPEC) cannot be defined by specific genetic signatures for pathotype characterization and are usually categorized by their site of isolation (uropathogenic *E. coli* or UPEC; neonatal meningitis-associated *E. coli* or NMEC; sepsis-related *E. coli* or SEPEC). On the other hand, the six distinct diarrheagenic *E. coli* (DEC) pathotypes are classified by their specific virulence factors, mechanisms of pathogenicity or adhesion patterns in cultured epithelial cells [11,12]. Enteroaggregative *E. coli* (EAEC) is one DEC pathotype that causes acute and persistent diarrheal diseases as a consequence of a strong adherence to the intestinal mucosa, enterotoxins/cytotoxins secretion, and inflammatory processes, affecting individuals of all ages globally [13,14,15,16,17,18,19]. Nataro et al. (1987) initially defined EAEC by classifying its adhesion pattern on HEp-2 cells as the aggregative adherence (AA) pattern [20]. Currently, EAEC strains are grouped as typical or atypical, based on the presence or absence of the *aggR* gene, respectively [11]. This gene, located in the pAA virulence plasmid, encodes an AraC family transcriptional regulator that controls various EAEC virulence genes [21].

In bacterial systems, protein secretion plays a pivotal role in nutrient acquisition, fimbriae and flagella biogenesis, secretion of virulence factors, drug efflux, and adaptation to diverse environments [22]. The Gram-negative type V secretion system, also known as the autotransporter (AT) pathway, comprises different protein families, including the serine protease autotransporters of Enterobacteriaceae, or SPATEs [23,24], characterized by the presence of a catalytic His/Asp/Ser triad containing the serine protease motif (GDSGS) in the passenger domain [25]. SPATEs are currently classified into two classes based on the phylogenetic analyses of the amino acid sequences of the passenger domains [24,26]: class I, including SPATEs with cytotoxic activities, such as Sat, Pet, EspC, EspP, and SigA [15,27,28,29,30]; and class II, including the immunomodulatory SPATEs, such as Pic, SepA, Vat, Hbp/Tsh, EpeA, and EatA [16,31,32,33,34,35]. EAEC strains often harbor SPATE-encoding genes in different combinations, but *sepA*, *pic*, *sigA*, and *sat* are the most prevalent [36,37,38,39,40]. Interestingly, *sat* and *pic* are also among the most commonly observed SPATE-encoding genes in ExPEC strains isolated from BSIs [41,42,43,44,45].

Several studies have reported the detection of UPEC strains with genotypic and/or phenotypic EAEC characteristics [46,47,48,49,50,51,52,53,54,55,56], and EAEC strains isolated from BSIs [41,48,57,58,59,60,61,62]. In a previous study, we characterized a large collection of *E. coli* strains isolated from the bloodstream, leading to the identification of the strain EC092. This strain harbored genetic markers associated with EAEC (*aggR*, *aatA*, *aaiA*, and *aaiG*), in addition to four SPATE-encoding genes (*pet*, *pic*, *sat*, and *sepA*) [41,63], indicating its hybrid pathogenic potential. In the present study, whole-genome sequencing and comparative genomics revealed that despite being isolated from an extraintestinal site, EC092 is closely related to EAEC strains, shedding light on its capacity to cause both extraintestinal and intestinal infections.

## 2. Results

### 2.1. EC092 Draft Genome

The assembled genome of EC092 was 5335.514 bases, with 1012-fold coverage based on the median *E. coli* genome size, or 973 times considering the EC092 draft genome size (Table S1). The draft genome has 473 contigs in which 5532 total CDSs, 5661 total genes, and 88 tRNAs were identified. The assembly statistics are shown in Table S2. Whole-genome sequences were deposited in the GenBank database under the accession number (GCA_024199065.1) and BioProject (PRJNA855471).

### 2.2. EC092 Genetic Features Provide Insights into Its Virulence

The pathogenesis of EAEC depends on various specific virulence factors, many of them identified in the prototypical EAEC 042 [64]. To date, at least three categories of adhesins that play a crucial role in EAEC strains have been identified: five different types of aggregative adherence fimbriae (AAF), the aggregate-forming pilus (AFP), and, more recently, a fibrillar adhesin known as CS22. Although EC092 carries the *aggR* gene, genes encoding AAFs, AFP, or CS22 were not detected.

We expanded our genomic search to investigate the presence of other virulence genes that define enteropathogenic *E. coli* (EPEC), Shiga toxin-producing *E. coli* (STEC), enterotoxigenic *E. coli* (ETEC), and enteroinvasive *E. coli* (EIEC). In fact, EC092 did not harbor any of the searched DEC virulence genes other than those related to EAEC. Various virulence factors were recognized as encoded by the annotated CDSs, such as adhesins, invasins, iron-uptake systems, bacteriocins, toxins, and genes involved in serum resistance. The Abricate tool, which includes multiple databases, including ecoli_VF, was used for the mass screening of virulence gene contigs (Table 1).

Finally, in silico analyses showed that EC092 belongs to the serotype O165:H4, phylogroup B1, and sequence type (ST) 278. Bacterial resistance involves various mechanisms that enable bacteria to survive the action of antibiotics. In the EC092 strain, genes conferring resistance to multiple groups of antibiotics, such as trimethoprim, tetracycline, streptomycin, and sulfamethoxazole, have been identified (Table S3). Furthermore, the presence of multiple efflux pump-encoding genes in EC092, including *acrA*, *acrB*, *acrD*, *acrE*, *acrF*, *mdtE*, *mdtP*, *mdtO*, *mdtN*, *tolC* (resistance–nodulation–division), *emrA*, *emrB*, *emrK*, *emrY*, *mdtM*, *mdfA*, *mdtG*, *mdtH*, *mdtF* (major facilitator superfamily), and *emrE* (small multidrug resistance), indicates a highly effective defense mechanism against various antibiotics. Efflux pumps not only expel antimicrobial compounds from the cell but are also essential in the formation and maintenance of biofilms [65]. Studies have shown that the presence of the *acrD*, *acrE*, *emrA*, *emrB*, and *emrE* genes is associated with a reduction in biofilm growth [66], which may explain the inability of EC092 to form a biofilm in polystyrene (Del Carpio, A.M.G., Butantan Institute, São Paulo, Brazil. Personal communication).

The amino acid sequences deduced from the nucleotide sequences of the detected SPATE-encoding genes were aligned and the main findings extracted from the alignments are shown in Table S4. The amino acid alignments in their full length are shown in Figures S1–S4. The alignments were also performed between the EC092 amino acid predicted sequences of AggR, AaiC, AatA, and Aap and their respective reference sequences from EAEC 042, finding a 100% similarity between the predicted amino acid sequences of these genes in EC092 and EAEC 042. The amino acid alignments in their full length are shown in Figures S5–S8.

### 2.3. Phylogenetic Analyses Show That EC092 Is Closely Related to Other EAEC Strains

In order to understand the phylogenetic relationships between EC092 and other *E. coli* strains, a phylogenetic tree was generated compiling a panel of representative genomes from different *E. coli* pathotypes, including ExPEC, EAEC, EPEC, STEC, enterohemorrhagic *E. coli* (EHEC), ETEC, EIEC, adherent-invasive *E. coli* (AIEC), and commensal *E. coli* strains (Table S5).

**Figure 1:**
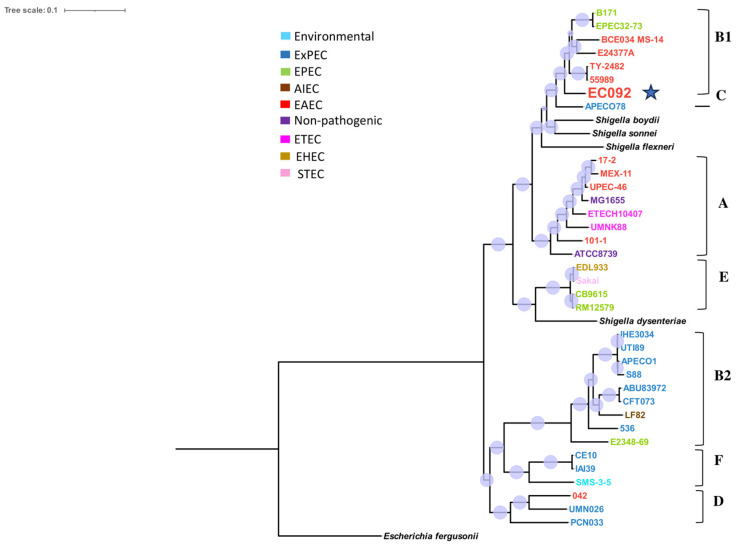
Phylogram of strain EC092 and various reference strains of *E. coli*. The phylogram, based on the core SNPs, was constructed using the KSNP4.0 software phylogenetic tree tool and the maximum likelihood phylogenetic reconstruction technique. The branch support was calculated based on the Shimodaira-Hasegawa (SH) test, where the dots in lilac denote 100% of reliability of the branch. The tree was created using the iTOL version 6.9 tool. Four strains of *Shigella* spp. and one strain of *Escherichia fergusonii* were used as the outgroups. The different groups of *E. coli* strains (commensal, environmental, EAEC, EPEC, STEC, ETEC, AIEC, and ExPEC) and phylogroups (A, B1, B2, C, D, E, and F) are represented in different colors. The EC092 strain is indicated by a blue star.

The resulting tree (Figure 1) showed that EC092 is closely related to the EAEC strains 55989 (GCA_000026245.1) and TY2482 (GCA_000217695.2) that belong to the B1 phylogroup, serotype O104:H4 and ST 678. Yet, the tree indicates that EC092 shares a common ancestor with the EAEC strains but not with the other strains of ExPEC. In addition to the completely different isolation sites, this analysis demonstrates that EC092 (isolated from the bloodstream) and EAEC (isolated from feces) share genetic characteristics and are more closely related to each other than EC092 is to the other strains selected for the analysis, including the ExPEC strains.

**Figure 2:**
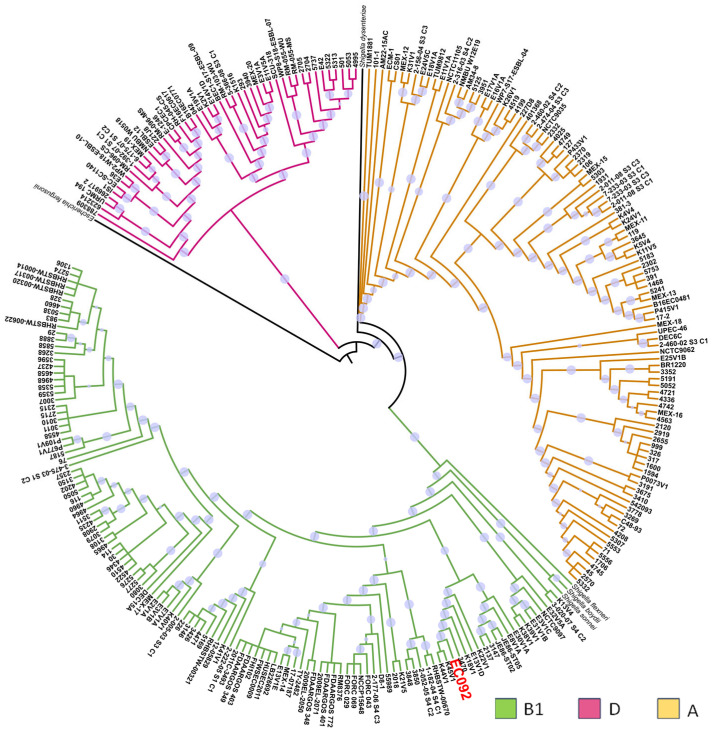
Phylogenetic tree based on EC092 and the other 270 EAEC strains’ genomes. The phylogram, based on the core SNPs, was constructed using the KSNP4.0 software phylogenetic tree tool and the maximum likelihood phylogenetic reconstruction technique. The branch support was calculated based on the SH test, where the dot in lilac denotes 100% reliability of the branch. The tree was created using the iTOL version 6.9 tool. Four strains of *Shigella* spp. and one strain of *E. fergusonii* were used as the outgroups. The strains are colored according to the phylogenetic group of *E. coli*. The EC092 strain is indicated in red color.

In a second phylogenetic analysis, the relationship between EC092 and the 270 genomes of EAEC belonging to various serotypes and STs was evaluated, including the genomes of four strains of *Shigella* spp. and one *E. fergusonii* as the external groups (Table S6). This analysis showed that most of the EAEC strains were distributed in three phylogenetic groups: A, B1, and D (Figure 2), and it is of note that EC092 is related to the EAEC strains that also belong to the B1 phylogenetic group, in a clade closely associated only with the EAEC strains that do not meet the criteria that define ExPEC intrinsic virulence, i.e., the presence of at least two of the following genes: *afa*/*dra*, *papA* and/or *papC*, *sfa*/*foc*, *iucD*/*iutA*, and *kpsMT* II [67]. The four EAEC strains most closely related to EC092 were isolated from diarrheic feces in Kenya (K44V1, K45V1, and K18V1) and Egypt (E13V1D) that belong to serotypes and STs that are different from the ones presented by EC092 [68]. While the K44V1 and K45V1 strains belong to the O61:H4 serotype and ST248, the K18V1 strain belongs to the O9:H2 serotype and ST155, and the E13V1D strain belongs to the ONT:H7 serotype and ST2707.

### 2.4. Virulence Factors Distribution Shows Different Patterns in Various EAEC

**Figure 3:**
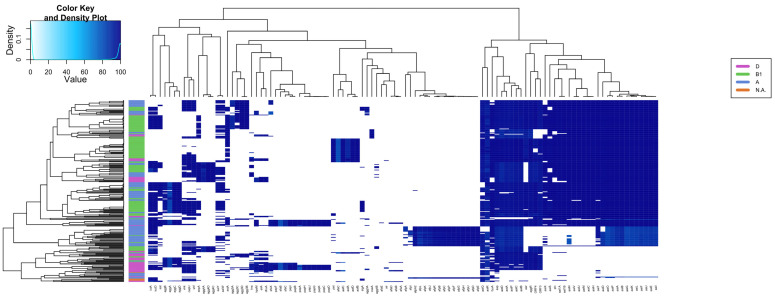
Heatmap and hierarchical clustering based on the presence of several virulence genes in EAEC genomes and EC092. The presence/absence of 116 virulence genes was assessed in 270 EAEC genomes and five outgroup strains (*Shigella* spp. and *E. fergussoni*), highlighting different patterns of genes distribution. The heatmap is colored according to the identity value shown in the top left box. The dendrograms (above and on the left side) correspond to the hierarchical clustering of the genes (above) and genomes (left) used.

The presence of 116 EAEC and ExPEC-related genes was investigated in the 270 genomes of EAEC, including EC092, resulting in specific groups based on the presence of such genes (Figure 3 and Table S7). A significant genetic similarity of EC092 with EAEC strains isolated from human feces, with similar virulence profiles, such as the presence of classical virulence genes of EAEC (*aggR*, *aatA*, and *aap*) was observed. EC092 also harbors a type 6 secretion system (T6SS) known as *aaiA-Y*, with only *aaiW* being absent. It is important to note that the genomes of the EAEC strains K45V1 and K44V1, isolated from the feces of patients with diarrhea, and the genome of EC092, isolated from bacteremia, harbor almost the same number of genes selected for the heatmap (Table S8).

This analysis also allowed us to determine the prevalence of the genes associated with ExPEC and UPEC in those EAEC genomes. Strain EC092 possesses only the *iutA* gene, related to extraintestinal virulence, and does not meet the ExPEC criteria established by Johnson et al. (2003), although it was isolated from the bloodstream. Regarding its potential uropathogenicity, the exclusive presence of the *fyuA* gene does not classify EC092 as UPEC [69]. This overall analysis revealed that EC092 is indeed a hybrid pathogenic lineage, i.e., EAEC/ExPEC.

Finally, the 270 EAEC genomes were evaluated for the presence of 710 genes encoding the main virulence factors of DEC and ExPEC, including those that encode iron-uptake genes (Figure S9 and Table S9), fimbria biogenesis (Figure S10 and Table S10), adhesion/invasion processes (Figure S11 and Table S11), autotransporter proteins (Figure S12 and Table S12), toxins (Figure S13 and Table S13), and type VI secretion systems (Figure S14 and Table S14). From the results of all the heatmaps, it was observed that the genomes of the EAEC clinical strains K44V1 and K45V1, isolated from the feces of military personnel with diarrhea [68], were closely related and showed a high genetic similarity of the virulence profile with the EC092 genome, except for some genes that encode toxins (*mchF*, *mchC*, *mchE*, *mcmK*, *cvaA*, *mcmM*, *mchD*, *mchB*, and *mchI*), type VI secretion systems (*yhhI-1*), autotransporters (*cah*), and adhesion and invasion processes (*elfD* and *elfA*) that were absent in K44V1 and K45V1.

### 2.5. Genetic Neighborhood Analysis Reveals a Close Relationship between the EC092 and EAEC Strains K44V1 and K45V1 Isolated from Feces

The genetic neighborhood encompassing some genes of interest was examined to gain further insights into the genetic associations and similarities between these strains. This analysis revealed that EC092 has a close relationship with the EAEC strains K44V1 and K45V1 isolated from feces, characterized by shared genetic features and organization, including the presence of genes such as *pic*, *pet*, *sat*, *sepA*, and *aggR*, and the operons *aatA-P*, *aaiA*, and *aap* in identical collinear blocks shared among these genomes (Figure S15).

### 2.6. Pet, Pic, Sat, and SepA Production by EC092

**Figure 4:**
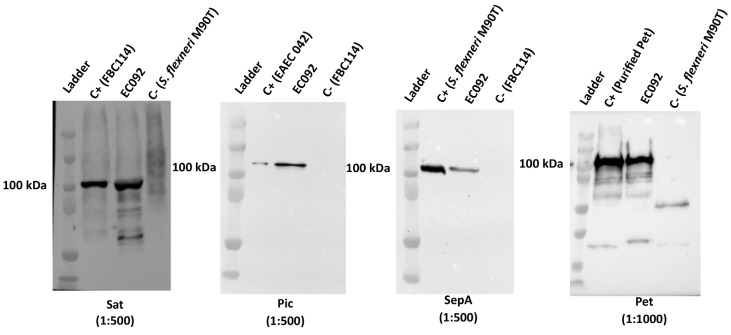
Detection of SPATEs in culture supernatants of EC092. Concentrated supernatants of EC092 and the respective positive (C+) and negative (C−) controls were analyzed using 8% sodium-dodecyl sulfate-polyacrylamide gel electrophoresis (SDS-PAGE), followed by immunoblotting using specific antisera. Ladder: Kaleidoscope™ Prestained Protein Standards (BioRad). Sat (107 kDa; anti-Sat: 1:500); Pic (116 kDa; anti-Pic: 1:500); SepA (110 kDa; anti-SepA: 1:500); Pet (104 kDa; anti-Pet: 1:1000). The reaction was developed employing a goat anti-rabbit IgG peroxidase conjugate (1:5000).

Considering that EC092 harbors *pet*, *pic*, *sat*, and *sepA*, we decided to investigate if the four respective proteases encoded by these genes were produced by this strain. EC092 culture supernatants were analyzed by immunoblotting using specific antisera and the reactivity with proteins of approximate size of 100 kDa, corresponding to Pet, Pic, Sat and SepA, were observed for EC092 (Figure 4). This indicates that all four SPATEs are produced and secreted in vitro by EC092.

### 2.7. E. coli EC092 Is Not Killed by Normal Human Serum (NHS)

**Figure 5:**
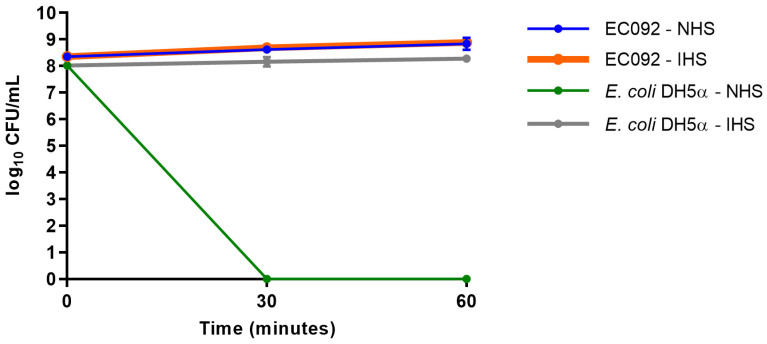
Resistance against the bactericidal activity of normal human serum. The CFU/mL counts of the EC092 and *E. coli* DH5α strains on MacConkey agar were obtained with their initial inoculum (corresponding to time zero) and after 30 min and 60 min of exposure to normal human serum (NHS) or heat-inactivated normal human serum (IHS), both at a concentration of 50%. The counts were analyzed using the GraphPad Prism software (version 7.00).

Although EC092 displayed EAEC features, this strain was isolated from BSI. Since the ability to survive the bactericidal activity of serum is a crucial feature displayed by bloodstream-isolated *E. coli*, the serum resistance of EC092 was assessed. As shown in Figure 5, EC092 survived in the presence of NHS, as no differences in CFU/mL were observed between NHS and heat-inactivated NHS (IHS). As expected, *E. coli* DH5α was killed within the first 30 min upon contact with NHS and survived completely in heat-inactivated NHS.

## 3. Discussion

EAEC has emerged in recent years as a pathogen associated with extraintestinal infections, which is evidenced by several reports of EAEC strains isolated from urinary tract infections [47,48,49,50,51,52,53,54,55,56], bacteremia, or sepsis [41,57,59,60,61,62,70]. EC092 has been selected among the *E. coli* strains isolated from bacteremia due to the presence of EAEC characteristics, such as the AA pattern on HEp-2 cells and the presence of *aggR*, *aatA*, *aaiC*, and *aap* [41,63]. Genotypic and phenotypic analyses of EC092 were performed in this study to evaluate its genome, leading to insights into its possible hybrid pathogenic potential.

Through the whole-genome sequencing of EC092, it was identified that various virulence factors are present in this strain, such as adhesins, invasins, iron-uptake systems, bacteriocins, toxins, and serum resistance-associated genes, and the presence of EAEC genetic markers (*aatA*, *aggR*, *aaiA*, *aaiG*, and *aap*) and SPATE-encoding genes (*pet*, *pic*, *sat*, and *sepA*) was also confirmed. The investigation of virulence genes associated with ExPEC showed that EC092 does not fit into the criterium proposed by Johnson et al. to detect *E. coli* strains possessing a genetic background that is able to cause extraintestinal infection in a healthy person [67]. Also, EC092 does not harbor the genes defining the uropathogenic potential of an *E. coli* strain [69]. These findings reinforce the classification of EC092 as an EAEC. Accordingly, cases of *E. coli* lacking intrinsic ExPEC virulence genes but that are still capable of causing extraintestinal infections have been reported [41,53,70,71,72,73].

It is important to highlight that, in addition to the close phylogenetic relation of EC092 with EAEC genomes (Figure 1), the presence of an aggregative adherence plasmid (pAA) is strongly indicated by the identification of the following genes located in the pAA2 (GenBank accession number: NC_017627.1) of the EAEC prototype 042: *pet*, *aar*, *aggR*, *aaiQ*, *aatAPBCD*, *orf3*, *orf4*, *shf*, *capU*, *virK*, and *aap* (Table S7). The pAA2 belongs to the IncFIIA family [64], characterized by the presence of the RepA replicon (Ec042_RS29825), which is also present in the genome of EC092 (GenBank accession GCA_024199065.1). Moreover, the presence of a high-molecular-weight plasmid (~100 kb) similar to pAA2 was detected in the plasmid profile analysis of EC092 (Figure S16).

None of the genes related to the biogenesis of the five AAF fimbriae variants were found in the genome of EC092 (Table S10), but other adhesin-encoding genes were identified as potential factors that could be mediating the AA phenotype of EC092, including ECP, Hra1, and LPF [74,75,76,77,78]. The role of these adhesins in EC092 epithelial colonization is currently under investigation.

The presence of four SPATE-encoding genes in EC092 is an uncommon characteristic in DEC or ExPEC strains [36,37,38,39,40,41,79,80,81]. SPATEs play a significant role in bacterial virulence, including biofilm formation, cytotoxicity, and immunomodulation [24]. Furthermore, Pet, Sat, Pic, and SepA were detected in the culture supernatant of strain EC092, indicating its high pathogenic potential in systemic infections. These serine proteases play a crucial role in cytotoxicity, cell invasion [82,83,84,85,86], and innate immune system evasion [42,87,88,89]. Therefore, one possible source for the bacteremia caused by EC092 could be related to the production of these SPATEs during intestinal colonization, leading to bacterial translocation to the bloodstream. Following this hypothesis, the cytotoxic activities mediated by Pet, Sat, and SepA could facilitate the translocation of EC092 from the intestinal lumen to the lamina propria, followed by Sat injuries to the capillary endothelium, facilitating access to the bloodstream. Once in the bloodstream, Pic, Pet, and Sat could cleave the complement system proteins and glycoproteins of leucocytes, mediating evasion of the innate immune system, leading to sepsis.

A phylogenetic tree analysis, based on the sequenced genome of EC092 in comparison with the genomes of various *E. coli* pathotypes (DEC and ExPEC), resulted in clustering EC092 with the EAEC strains 55989 (AAF/III prototype), C227-11 (Stx-producing EAEC), and TY2482 (Stx-producing EAEC). These results clearly indicate that EC092 has a significant genetic proximity to the B1 phylogroup–EAEC strains and is less genetically related to the ExPEC strains. Considering that some bacterial pathotypes are frequently associated with specific phylogroups [90,91] we conducted a second phylogenetic analysis, using the genome of EC092 in comparison with the 270 genomes of EAEC belonging to different serotypes and sequence types (STs). The EAEC genomes analyzed in the second phylogenetic tree of this study encompassed various STs, including ST10, ST38, ST40, ST131, and ST678, all of which have been associated with diseases or identified in diarrheal outbreaks [47,52,67,92,93]. Remarkably, the discrepancy observed regarding the serotype of the strains composing the phylogenetic cluster with EC092 also extends to the ST, i.e., ST278 was exclusively identified in EC092.

To investigate the prevalence of the characteristic EAEC, ExPEC, and UPEC genes among the EAEC genomes, we constructed a heatmap analysis comparing 116 genes and 270 EAEC genomes collected from the GenBank database. In the first heatmap, we examined an extensive collection of EAEC strains from various geographical regions. All these EAEC genomes shared at least one gene regulated by AggR. Strains harboring the *aggR* gene are known as typical EAEC, while those lacking this gene are termed atypical [11]. According to this classification, strain EC092 is classified as a typical EAEC, as it possesses genes such as *aggR*, *aatA*, *aap*, *aaiA*, and *aaiC*, in addition to exhibiting the AA pattern of adherence to HEp-2 cells [63].

Furthermore, additional heatmaps were generated, enabling the analysis of various virulence genes among a large collection of EAEC genomes, including EC092 for comparative purposes.

A heatmap analysis of the genes encoding proteins involved in iron acquisition revealed that all the EAEC genomes contained the genes *fepABC*, *fes*, *entABCDEF*, *fhuD*, and *feoAC* (Figure S9 and Table S9). Additionally, the EAEC genomes from the phylogroup D possessed the complete *chuAVYSWTXU* operon. The EC092 strain harbored the genes *irp2*, *fes*, *shiF*, and *cirA*, and the complete operons *fepABCDEG*, *entABCEFDS*, *feoABC*, *iucABCD*, *ybtAEPSTQXU*, *fecABCDEIR*, *efeBOU*, and *fhuABCDF*, but lacked the *fhuE* gene.

Considering the presence of adhesion/invasion genes, EC092 showed proximity to genomes with a large number of fimbriae-encoding genes, including the operons *yadKLMVN*, *yehEDCAB*, *yfcUSV*, *ybgPOQ*, *sfmFACHD*, *yraNOQPRLJHIK*, and *eafABCD* (Figure S10 and Table S10), as well as the genes *htrE* and *focA_2*, conferring adhesion and pathogenicity capabilities. The presence of the genes *lpfABCD*, *hra1*, *shf*, *elf*, and *elfC*, and the operons *ecpABCDE*, *aatABCDP*, and *csgABCDEFG* (Figure S11 and Table S11), in addition to the *aap* and *hcp* genes, indicates a significant role in biofilm formation, toxin secretion, and immune evasion. Among the autotransporter genes, besides *sepA*, *pet*, *pic*, and *sat*, the genes *eha*G, *cah*, *yejA*, *ehaC*, and *yfaL* were found (Figure S12 and Table S12). The toxin-encoding genes in EC092 include *hlyE* and *mcmL* (Figure S13 and Table S13), and the genes encoding the SST6 system comprise *vrgG*, *aec27*, *aec26*, *aec28*, *ets*, *yhhI-1*, *icmF*, *impA*, and the *aai* operon (Figure S14 and Table S14). An analysis of all the heatmaps, the EAEC phylogeny (Figure 2), and the shared genomic organization (Figure S15) suggests that EC092 is related to the K44V1 and K45V1 genomes due to highly shared genetic characteristics and structure [68].

Hybrid pathogenic *E. coli* strains include those harboring virulence genetic markers that are typical of DEC and ExPEC pathotypes, or are isolated from extraintestinal infections and possess DEC virulence markers [72]. Therefore, the results obtained in the study enabled the classification of EC092 as a hybrid EAEC/ExPEC strain. Several authors have described the presence of hybrid DEC/ExPEC strains in patients, notably those associated with cases of bacteremia, such as the example of the O80:H2 serotype STEC/ExPEC strain [94]. The sequencing of a hybrid EPEC/ExPEC strain, isolated from a patient with severe prolonged diarrhea, bacteremia, and multiple organ dysfunction, revealed that it was, in fact, an ExPEC strain that also had distant orthologous genes from the typical EPEC genes [95]. Similarly, reports in the literature also highlight EAEC strains isolated in sepsis [41,48,57,59,60,61,62,70].

Based on the phylogenetic similarities observed between strain EC092 and the EAEC strains K44V1 and K45V1 (isolated from the feces of patients with diarrhea) [68], considering that EAEC is recognized as a primarily intestinal pathogen, the translocation of EC092 from the intestinal mucosa to the bloodstream could have been a possible route to establish the bloodstream infection.

To our knowledge, this study represents the first in the literature to investigate the complete genome and virulence factor arsenal of an EAEC strain isolated from a case of BSI. The analyses conducted here can provide valuable insights for future epidemiological investigations and clinical characterizations of EAEC involved in extraintestinal infections. The genome of strain EC092 constitutes an intriguing model of a hybrid pathogenic lineage. The diversity of virulence genes present in the genome of EC092, including those that mediate colonization, toxicity, and evasion of the innate immune system, suggests the hybrid pathogenic potential of this strain, i.e., the possibility of causing both intestinal and extraintestinal infections. Additionally, in vivo tests using different animal models are underway to validate a hypothetical model of how EC092 causes extraintestinal infection. This will provide a more comprehensive understanding of virulence mechanisms and enhance the development of more effective therapeutic strategies.

## 4. Materials and Methods

### 4.1. Bacterial Strains

EC092 was isolated from blood culture at the University Hospital of the Federal University of São Paulo (Hospital São Paulo, HSP-UNIFESP) in São Paulo, Brazil. The strain is part of the Enterobacterales–Extraintestinal collection (EPM-DMIP) maintained by the Department of Microbiology, Immunology and Parasitology of the Federal University of São Paulo (UNIFESP). This strain harbors four SPATE-encoding genes (*pet*, *sat*, *pic*, and *sepA*), belongs to phylogroup B1, lacks genetic markers defining the potential to cause extraintestinal infections, produces the AA pattern on HEp-2 cells, and harbors *aggR*, *aatPABCD*, *aaiA*, and *aaiG* genes [41,63].

The following bacterial strains were used as controls in immunoblots for SPATEs detection: EAEC 042, *Shigella flexneri* 5a M90T, diffusely adherent *E. coli* FBC114, *E. coli* HB101, and *E. coli* DH5α [64,96,97,98].

### 4.2. DNA Extraction, Library Sequencing, and Read Filtering

EC092 was grown in Lysogeny Broth (LB) (Difco, Omagh, UK) for 18 h at 37 °C for DNA extraction, which was carried out using the QIAamp^®^ DNA Mini kit (Qiagen, Merck, Germany). The DNA concentration was determined using the PicoGreen kit (ThermoFisher Scientific, Waltham, MA, USA) and assessed for its integrity using a microvolume electrophoresis instrument (Bioanalyzer, Agilent, Santa Clara, CA, USA). All the procedures were carried out following the manufacturer’s instructions. The whole genome was sequenced using the HiSeq 1500^®^ System (Illumina, San Diego, CA, USA) platform at the Applied Toxinology Laboratory of Butantan Institute, employing a 250 bp paired-end protocol. The sequences were pre-processed using the Casava 1.6 software (Illumina) and analyzed for read quality using the FastQC (http://www.bioinformatics.babraham.ac.uk/projects/fastqc (accessed on 2 July 2021)) and MultiQC (https://multiqc.info/ (accessed on 11 January 2022)) tools. The sequences were processed to remove low-quality bases, small reads, adapters, and contaminants using the Fastp [99], AdapterRemoval [100], and Bowtie2 [101] tools, respectively.

### 4.3. Assembly, Annotation, and Screening for Known Virulence Factors

The EC092 genome was assembled using the SPAdes software version 3.12.0 [102], and only contigs ≥ 200 bp were retained. The assembled genome was then analyzed using the QUAST tool [103] and aligned against the EAEC 042 reference (GenBank accession GCA_000027125.1). EC092 complete genome sequences were annotated using Prokka version 1.13.3 and deposited in the GenBank: GCA_024199065.1 and BioProject PRJNA855471.

The EC092 genome was submitted to the Center for Genomic Epidemiology (CGE) tools to determine its sequence type (multilocus sequence typing—MLST), serotype (SerotypeFinder), and resistance profile (ResFinder). The presence of virulence genes was analyzed using the ecoli_VF v0.1 in the ABRicate tool (https://github.com/tseemann/abricate (accessed on 29 July 2021)). The EC092 phylogenetic classification was determined using the ClermontTyping tool [104]. The analysis of the SPATE-coding genes was carried out by aligning the EC092 DNA sequences with the reference DNA sequences obtained in GenBank—NCBI (National Center for Biotechnological Information), using the BLASTn program.

### 4.4. Phylogenetic Relationships of EC092 with Different Groups of E. coli

A phylogenetic tree including strain EC092 was constructed using the KSNP4.0 software phylogenetic tree tool [105], using core SNPs and the maximum likelihood phylogenetic reconstruction technique. The KSNP4.0 tool uses FastTree to infer the phylogeny as well to estimate the branch support values, based on the Shimodaira–Hasegawa test (SH test) with 1,000 bootstrap replicates [106].

To establish the phylogenetic relationships, a total of 34 reference *E. coli* strains from GenBank were used for comparison: 12 ExPEC (APEC078, PCN033, APEC01, IAI39, UTI89, ABU83972, 536, UMN026, CFT073, S88, IHE3034, and CE10), one environmental (SMS-3-5), two nonpathogenic (ATCC 8739 and K-12 MG1655), three ETEC (H10407, UMNK88, and E24377A), one EHEC (EDL933), one STEC (Sakai), five EPEC (E2348/69, CB9615, RM12579, B171, and EPEC 32-73), one AIEC (LF82), and eight EAEC (042, 55989, 101-1, BCE034_MS-14, TY_2482, MEX-11, UPEC-46, and 17-2). Also, four *Shigella* spp. (*S. boydii*, *S. sonnei*, *S. flexneri*, and *S. dysenteriae)* and *E. fergusonii* (ATCC 35469) were used as the outgroups (Table S5).

A second phylogenetic analysis was performed using the same method to evaluate the relationship between EC092 and the 270 representative EAEC strains belonging to different phylogenetic lineages and MLSTs, and four *Shigella* spp. (*S. boydii*, *S. sonnei*, *S. flexneri*, and *S. dysenteriae*) and *E. fergusonii* ATCC 35469 were used as the external groups (Table S6). Both trees were visualized using the iTOL version 6.9 tool.

### 4.5. Presence and Absence of Virulence Factors in Different EAEC Strains

The EC092 and 270 EAEC reference genomes were grouped into a variety of *E. coli* genomes (Tables S6 and S8) using the hierarchical clustering method, based on the identity of virulence factors. Databases containing the nucleotide sequences of genes coding for toxins, secretion systems, iron-acquisition systems, autotransporter proteins, adhesins, and invasins were specifically created based on ecoli_VF (Table S8). The alignment was carried out using Blastn [107] with the default parameters, and the data were analyzed using the open-source programming language R [108], using the gplots package to create heatmaps. Finally, the GIMP 2.10.32 program was used for editing the heatmap images.

### 4.6. Genomic Neighborhood

To evaluate the collinearity of some blocks of genomes from the strains EC092, K44V1, and K45V1 and to confirm the orthology of the genes *aggR*, *aap*, *pet*, *pic*, *sat*, and *sepA*, as well as the operons *aat* and *aai*, among these genomes, the genomic neighborhoods that surround these genes were examined for all these strains. For the visualization and creation of the linear comparison figures, the Easyfig tool [109] was used.

### 4.7. Detection of Pet, Pic, Sat, and SepA in the EC092 Culture Supernatant

The proteins present in the supernatants of strains EC092, EAEC 042 (Pet- and Pic-producer), *S. flexneri* M90T (SepA-producer), and DAEC FBC114 (Sat-producer) were precipitated using trichloroacetic acid (TCA, Sigma-Aldrich, St. Louis, MO, USA) [110]. The strains were cultured in LB for 18 h at 37 °C with constant shaking at 250 rpm. After this period, the cultures were centrifuged at 2000× *g* for 15 min at 4 °C, and 1 mL aliquots of the supernatants were subjected to precipitation with 20% TCA. Subsequently, the precipitates were obtained using centrifugation at 16,000× *g* for 15 min, followed by washes with acetone. The resulting precipitates were resuspended in Tris-HCl 1 M, at pH 8.8, and stored at −20 °C.

The samples were analyzed using SDS-PAGE [111]. Two gels were run under the same conditions. One was stained with a silver nitrate solution [112], and the other was used to be transferred to a nitrocellulose membrane for immunoblotting using specific polyclonal antisera.

Antisera against Pet, Pic, and Sat were obtained in previous studies [86,113,114]. A specific antiserum against SepA was obtained following the protocol approved by the Ethics Committee on Animal Use of the Butantan Institute (CEUAIB Protocol # 1395/15). One New Zealand rabbit (2.5 kg) was supplied by the Animal Research Facilities of the Butantan Institute. The rabbit was intramuscularly immunized with a 1 mL solution containing 100 μg of SepA and 2.5 mg of aluminum hydroxide as an adjuvant. The immunization schedule included three doses with a 15-day interval between each administration. A pre-immune serum sample was collected before the first immunization, and after the initial immunization, a subsequent blood sample was collected to obtain the immune serum. Both pre-immune and immune sera were employed for the SepA serum titration. After 45 days from the second immunization, the rabbit was euthanized to collect the hyperimmune serum. The blood was incubated at 37 °C for 30 min, and centrifuged at 180× *g* for 10 min at 4 °C. The collected serum was incubated at 56 °C for 30 min and stored at −20 °C.

The nitrocellulose membranes were incubated in a blocking solution (5% skimmed milk in 0.01 M PBS), followed by incubation with specific polyclonal antibodies against Sat (1:500), SepA (1:500), Pic (1:500), or Pet (1:1000) for 2 h, with shaking at room temperature. After the washes, the membrane was incubated with a goat anti-rabbit IgG antibody peroxidase conjugate (1:5000). Signal detection was achieved using the SuperSignal^®^ West Pico Enhanced Chemiluminescent Substrate and the Alphaimager imaging system.

### 4.8. Resistance against the Bactericidal Activity of Human Serum

The resistance capacity of EC092 to the bactericidal effects of normal human serum (NHS) was assessed as previously described [23,115]. Initially, both EC092 and *E. coli* DH5α were cultivated in 50 mL of LB with continuous shaking (250 rpm) at 37 °C until they reached the OD of 0.5 at λ = 600 nm.

Duplicate sets of 100 µL of NHS (Sigma-Aldrich) were mixed with 80 µL of sterile 0.01 M PBS for each bacterial strain. The first set of tubes was incubated at 37 °C for 30 min, while the second set was subjected to heat inactivation at 56 °C for 30 min. Additionally, a third set containing 180 µL of sterile 0.01 M PBS was used as a control and was also incubated at 37 °C for 30 min. Subsequently, 20 µL of the bacterial inoculum were added to each tube. The tubes containing NHS and heat-inactivated NHS were then incubated at 37 °C. After 30 min and 60 min of incubation, 20 µL from each time point were collected, serially diluted, and plated onto MacConkey agar plates. Subsequently, the plates were incubated at 37 °C for 18 h for colony-forming unit (CFU) counting. The statistical analysis involved comparing the results of each tested condition at different incubation periods using the ANOVA and Tukey’s multiple comparison tests, with 95% confidence intervals.

## Figures and Tables

**Table 1:** Virulence factors identified in the genome of EC092.

Groups ^a^	Gene	Name
Virulence regulator	*aggR*	Transcriptional activator
Adhesion and invasion	*aap*	Dispersin, anti-aggregation Protein
	*aatABCDP*	Dispersin transporter
Fimbriae	*ecpABCDER*	*E. coli* common pilus
	*ycbFSTUV*	Ycb fimbria
	*csgABCDEFG*	Curli
Iron acquisition	*entABCDEFS*	Enterobactin
	*fepABCDEG*	Ferroenterobactin
	*fes*	Ferric enterobactin esterase
	*fyuA*	Siderophore receptor
	*irp2*	Yersiniabactin biosynthetic system
	*iucABCD*	Aerobactin synthetase
	*iutA*	Ferric aerobactin receptor
Type 2 secretion system	*gspCDEFGHIJKLM*	T2SS-1
Type 3 secretion system 2	*ygeGH*	ETT2
	*eprHIJK*	ETT2
	*epaOPQRS*	ETT2
Type 6 secretion system	*aaiABCDEFGHIJKLMNOPX*	AggR-activated island
Type 5 secretion system	*pet*	Pet
	*pic*	Pic
	*sat*	Sat
	*sepA*	SepA
	*upaG*/*ehaG*	UPEC autotransporter
	*cah*	Cah
	*ehaC*	EHEC autotransporter
Toxins	*hlyE*	Hemolysin E
Flagellum	*flgEFGHIJKL*	Flagellar protein
	*fliAFGIJMPR*	Flagellar protein
	*flhAB*	Flagellar protein
Serum resistance	*traT*	Serum survival

^a^, Groups of genes as classified by the ecoli_VF database.

## Data Availability

The data supporting the findings of this study are available within the article and its Supplementary Materials. The NCBI accession number of the EC092 genome sequence is GCA_024199065.1, BioProject PRJNA855471, and JANAKE000000000.1, found at https://www.ncbi.nlm.nih.gov/. The raw data used in our analyses are available in the Butantan Institute Repository (https://repositorio.butantan.gov.br/handle/butantan/5379).

## Supplementary Materials

The supporting information can be downloaded at: https://www.mdpi.com/article/10.3390/ijms25179238/s1.

## References

[1] Laupland K.B., Church D.L. (2014). Population-Based Epidemiology and Microbiology of Community-Onset Bloodstream Infections. Clin. Microbiol. Rev..

[2] Kennedy K.J., Roberts J.L., Collignon P.J. (2008). *Escherichia coli* Bacteraemia in Canberra: Incidence and Clinical Features. Med. J. Aust..

[3] Laupland K.B., Gregson D.B., Church D.L., Ross T., Pitout J.D.D. (2008). Incidence, Risk Factors and Outcomes of *Escherichia coli* Bloodstream Infections in a Large Canadian Region. Clin. Microbiol. Infect..

[4] Al-Hasan M.N., Lahr B.D., Eckel-Passow J.E., Baddour L.M. (2009). Antimicrobial Resistance Trends of *Escherichia coli* Bloodstream Isolates: A Population-Based Study, 1998–2007. J. Antimicrob. Chemother..

[5] Williamson D.A., Lim A., Wiles S., Roberts S.A., Freeman J.T. (2013). Population-Based Incidence and Comparative Demographics of Community-Associated and Healthcare-Associated *Escherichia coli* Bloodstream Infection in Auckland, New Zealand, 2005–2011. BMC Infect. Dis..

[6] Johnson J.R., Russo T.A. (2005). Molecular Epidemiology of Extraintestinal Pathogenic (Uropathogenic) *Escherichia coli*. Int. J. Med. Microbiol..

[7] Scheutz F., Strockbine N.A. (2005). Genus I. Escherichia Castellani and Chalmers 1919, 941T. Bergey’s Man. Syst. Bacteriol..

[8] Tenaillon O., Skurnik D., Picard B., Denamur E. (2010). The Population Genetics of Commensal *Escherichia coli*. Nat. Rev. Microbiol..

[9] Clements A., Young J.C., Constantinou N., Frankel G. (2012). Infection Strategies of Enteric Pathogenic *Escherichia coli*. Gut Microbes.

[10] Croxen M.A., Finlay B.B. (2010). Molecular Mechanisms of *Escherichia coli* Pathogenicity. Nat. Rev. Microbiol..

[11] Kaper J.B., Nataro J.P., Mobley H.L.T. (2004). Pathogenic *Escherichia coli*. Nat. Rev. Microbiol..

[12] Johnson J.R., Russo T.A. (2018). Molecular Epidemiology of Extraintestinal Pathogenic *Escherichia coli*. EcoSal Plus.

[13] Bouckenooghe A.R., Dupont H.L., Jiang Z.D., Adachi J., Mathewson J.J., Verenkar M.P., Rodrigues S., Steffen R. (2000). Markers of Enteric Inflammation in Enteroaggregative *Escherichia coli* Diarrhea in Travelers. Am. J. Trop. Med. Hyg..

[14] Okhuysen P.C., DuPont H.L. (2010). Enteroaggregative *Escherichia coli* (EAEC): A Cause of Acute and Persistent Diarrhea of Worldwide Importance. J. Infect. Dis..

[15] Eslava C., Navarro-García F., Czeczulin J.R., Henderson I.R., Cravioto A., Nataro J.P. (1998). Pet, an Autotransporter Enterotoxin from Enteroaggregative *Escherichia coli*. Infect. Immun..

[16] Henderson I.R., Czeczulin J., Eslava C., Noriega F., Nataro J.P. (1999). Characterization of Pic, a Secreted Protease of Shigella Flexneri and Enteroaggregative *Escherichia coli*. Infect. Immun..

[17] Harrington S.M., Strauman M.C., Abe C.M., Nataro J.P. (2005). Aggregative Adherence Fimbriae Contribute to the Inflammatory Response of Epithelial Cells Infected with Enteroaggregative *Escherichia coli*. Cell. Microbiol..

[18] Navarro-Garcia F., Elias W.P. (2011). Autotransporters and Virulence of Enteroaggregative *E. coli*. Gut Microbes.

[19] Jensen B.H., Olsen K.E.P., Struve C., Krogfelt K.A., Petersen A.M. (2014). Epidemiology and Clinical Manifestations of Enteroaggregative *Escherichia coli*. Clin. Microbiol. Rev..

[20] Nataro J.P., Kaper J.B., Robins-Browne R., Prado V., Vial P., Levine M.M. (1987). Patterns of Adherence of Diarrheagenic *Escherichia coli* to HEp-2 cells. Pediatr. Infect. Dis. J..

[21] Morin N., Santiago A.E., Ernst R.K., Guillot S.J., Nataro J.P. (2013). Characterization of the AggR Regulon in Enteroaggregative *Escherichia coli*. Infect. Immun..

[22] Dautin N., Bernstein H.D. (2007). Protein Secretion in Gram-Negative Bacteria via the Autotransporter Pathway. Annu. Rev. Microbiol..

[23] Henderson I.R., Navarro-Garcia F., Nataro J.P. (1998). The Great Escape: Structure and Function of the Autotransporter Proteins. Trends Microbiol..

[24] Navarro-Garcia F. (2023). Serine Proteases Autotransporter of Enterobacteriaceae: Structures, Subdomains, Motifs, Functions, and Targets. Mol. Microbiol..

[25] Dautin N. (2010). Serine Protease Autotransporters of Enterobacteriaceae (SPATEs): Biogenesis and Function. Toxins.

[26] Ruiz-Perez F., Nataro J.P. (2014). Bacterial Serine Proteases Secreted by the Autotransporter Pathway: Classification, Specificity, and Role in Virulence. Cell. Mol. Life Sci..

[27] Al-Hasani K., Rajakumar K., Bulach D., Robins-Browne R., Adler B., Sakellaris H. (2001). Genetic Organization of the She Pathogenicity Island in Shigella Flexneri 2a. Microb. Pathog..

[28] Guyer D.M., Henderson I.R., Nataro J.P., Mobley H.L.T. (2000). Identification of Sat, an Autotransporter Toxin Produced by Uropathogenic *Escherichia coli*. Mol. Microbiol..

[29] Djafari S., Ebel F., Deibel C., Krämer S., Hudel M., Chakraborty T. (1997). Characterization of an Exported Protease from Shiga Toxin-producing *Escherichia coli*. Mol. Microbiol..

[30] Mellies J.L., Navarro-Garcia F., Okeke I., Frederickson J., Nataro J.P., Kaper J.B. (2001). EspC Pathogenicity Island of Enteropathogenic *Escherichia coli* Encodes an Enterotoxin. Infect. Immun..

[31] Benjelloun-Touimi Z., Si Tahar M., Montecucco C., Sansonetti P.J., Parsot C. (1998). SepA, the 110 KDa Protein Secreted by Shigella Flexneri: Two-Domain Structure and Proteolytic Activity. Microbiology.

[32] Parreira V.R., Gyles C.L. (2003). A Novel Pathogenicity Island Integrated Adjacent to the ThrW TRNA Gene of Avian Pathogenic *Escherichia coli* Encodes a Vacuolating Autotransporter Toxin. Infect. Immun..

[33] Provence D.L., Curtiss R. (1994). Isolation and Characterization of a Gene Involved in Hemagglutination by an Avian Pathogenic *Escherichia coli* Strain. Infect. Immun..

[34] Leyton D.L., Sloan J., Hill R.E., Doughty S., Hartland E.L. (2003). Transfer Region of PO113 from Enterohemorrhagic *Escherichia coli*: Similarity with R64 and Identification of a Novel Plasmid-Encoded Autotransporter, EpeA. Infect. Immun..

[35] Patel S.K., Dotson J., Allen K.P., Fleckenstein J.M. (2004). Identification and Molecular Characterization of EatA, An Autotransporter Protein of Enterotoxigenic *Escherichia coli*. Infect. Immun..

[36] Boisen N., Ruiz-Perez F., Scheutz F., Krogfelt K.A., Nataro J.P. (2009). Short Report: High Prevalence of Serine Protease Autotransporter Cytotoxins among Strains of Enteroaggregative *Escherichia coli*. Am. J. Trop. Med. Hyg..

[37] Boisen N., Scheutz F., Rasko D.A., Redman J.C., Persson S., Simon J., Kotloff K.L., Levine M.M., Sow S., Tamboura B. (2012). Genomic Characterization of Enteroaggregative *Escherichia coli* from Children in Mali. J. Infect. Dis..

[38] Andrade F.B., Abreu A.G., Nunes K.O., Gomes T.A.T., Piazza R.M.F., Elias W.P. (2017). Distribution of Serine Protease Autotransporters of Enterobacteriaceae in Typical and Atypical Enteroaggregative *Escherichia coli*. Infect. Genet. Evol..

[39] Havt A., Lima I.F., Medeiros P.H., Clementino M.A., Santos A.K., Amaral M.S., Veras H.N., Prata M.M., Lima N.L., Di Moura A. (2017). Prevalence and Virulence Gene Profiling of Enteroaggregative *Escherichia coli* in Malnourished and Nourished Brazilian Children. Diagn. Microbiol. Infect. Dis..

[40] Dias R.C.B., Tanabe R.H.S., Vieira M.A., Cergole-Novella M.C., dos Santos L.F., Gomes T.A.T., Elias W.P., Hernandes R.T. (2020). Analysis of the Virulence Profile and Phenotypic Features of Typical and Atypical Enteroaggregative *Escherichia coli* (EAEC) Isolated From Diarrheal Patients in Brazil. Front. Cell. Infect. Microbiol..

[41] Freire C.A., Santos A.C.M., Pignatari A.C., Silva R.M., Elias W.P. (2020). Serine Protease Autotransporters of Enterobacteriaceae (SPATEs) Are Largely Distributed among *Escherichia coli* Isolated from the Bloodstream. Braz. J. Microbiol..

[42] Freire C.A., Silva R.M., Ruiz R.C., Pimenta D.C., Bryant J.A., Henderson I.R., Barbosa A.S., Elias W.P. (2022). Secreted Autotransporter Toxin (Sat) Mediates Innate Immune System Evasion. Front. Immunol..

[43] Parham N.J., Srinivasan U., Desvaux M., Foxman B., Marrs C.F., Henderson I.R. (2004). PicU, a Second Serine Protease Autotransporter of Uropathogenic *Escherichia coli*. FEMS Microbiol. Lett..

[44] Ananias M., Yano T. (2008). Serogroups and Virulence Genotypes of *Escherichia coli* Isolated from Patients with Sepsis. Braz. J. Med. Biol. Res..

[45] Skjøt-Rasmussen L., Ejrnæs K., Lundgren B., Hammerum A.M., Frimodt-Møller N. (2012). Virulence Factors and Phylogenetic Grouping of *Escherichia coli* Isolates from Patients with Bacteraemia of Urinary Tract Origin Relate to Sex and Hospital- vs. Community-Acquired Origin. Int. J. Med. Microbiol..

[46] Abe C.M., Salvador F.A., Falsetti I.N., Vieira M.A.M., Blanco J., Blanco J.E., Blanco M., MacHado A.M.O., Elias W.P., Hernandes R.T. (2008). Uropathogenic *Escherichia coli* (UPEC) Strains May Carry Virulence Properties of Diarrhoeagenic *E. coli*. FEMS Immunol. Med. Microbiol..

[47] Boll E.J., Struve C., Boisen N., Olesen B., Stahlhut S.G., Krogfelt K.A. (2013). Role of Enteroaggregative *Escherichia coli* Virulence Factors in Uropathogenesis. Infect. Immun..

[48] Lara F.B.M., Nery D.R., de Oliveira P.M., Araujo M.L., Carvalho F.R.Q., Messias-Silva L.C.F., Ferreira L.B., Faria-Junior C., Pereira A.L. (2017). Virulence Markers and Phylogenetic Analysis of *Escherichia coli* Strains with Hybrid EAEC/UPEC Genotypes Recovered from Sporadic Cases of Extraintestinal Infections. Front. Microbiol..

[49] Modgil V., Kaur H., Mohan B., Taneja N. (2020). Molecular, Phylogenetic and Antibiotic Resistance Analysis of Enteroaggregative *Escherichia coli*/Uropathogenic *Escherichia coli* Hybrid Genotypes Causing Urinary Tract Infections. Indian J. Med. Microbiol..

[50] Nascimento J.A.S., Santos F.F., Santos-Neto J.F., Trovão L.O., Valiatti T.B., Pinaffi I.C., Vieira M.A.M., Silva R.M., Falsetti I.N., Santos A.C.M. (2022). Molecular Epidemiology and Presence of Hybrid Pathogenic *Escherichia coli* among Isolates from Community-Acquired Urinary Tract Infection. Microorganisms.

[51] Nazemi A., Mirinargasi M., Merikhi N., Sharifi S.H. (2011). Distribution of Pathogenic Genes AatA, Aap, AggR, among Uropathogenic *Escherichia coli* (UPEC) and Their Linkage with StbA Gene. Indian J. Microbiol..

[52] Olesen B., Scheutz F., Andersen R.L., Menard M., Boisen N., Johnston B., Hansen D.S., Krogfelt K.A., Nataro J.P., Johnson J.R. (2012). Enteroaggregative *Escherichia coli* O78:H10, the Cause of an Outbreak of Urinary Tract Infection. J. Clin. Microbiol..

[53] Toval F., Köhler C.D., Vogel U., Wagenlehner F., Mellmann A., Fruth A., Schmidt M.A., Karch H., Bielaszewska M., Dobrindt U. (2014). Characterization of *Escherichia coli* Isolates from Hospital Inpatients or Outpatients with Urinary Tract Infection. J. Clin. Microbiol..

[54] Yousefipour M., Rezatofighi S.E., Ardakani M.R. (2023). Detection and Characterization of Hybrid Uropathogenic *Escherichia coli* Strains among *E. coli* Isolates Causing Community-Acquired Urinary Tract Infection. J. Med. Microbiol..

[55] Tanabe R.H.S., Dias R.C.B., Orsi H., de Lira D.R.P., Vieira M.A., Dos Santos L.F., Ferreira A.M., Rall V.L.M., Mondelli A.L., Gomes T.A.T. (2022). Characterization of Uropathogenic *Escherichia coli* Reveals Hybrid Isolates of Uropathogenic and Diarrheagenic (UPEC/DEC) *E. coli*. Microorganisms.

[56] Moazeni S., Askari Badouei M., Hashemitabar G., Rezatofighi S.E., Mahmoodi F. (2024). Detection and Characterization of Potentially Hybrid Enteroaggregative *Escherichia coli* (EAEC) Strains Isolated from Urinary Tract Infection. Braz. J. Microbiol..

[57] Boll E.J., Overballe-Petersen S., Hasman H., Roer L., Ng K., Scheutz F., Hammerum A.M., Dungu A., Hansen F., Johannesen T.B. (2020). Emergence of Enteroaggregative *Escherichia coli* within the ST131 Lineage as a Cause of Extraintestinal Infections. mBio.

[58] Flament-Simon S.C., Nicolas-Chanoine M.H., García V., Duprilot M., Mayer N., Alonso M.P., García-Meniño I., Blanco J.E., Blanco M., Blanco J. (2020). Clonal Structure, Virulence Factor-Encoding Genes and Antibiotic Resistance of *Escherichia coli*, Causing Urinary Tract Infections and Other Extraintestinal Infections in Humans in Spain and France during 2016. Antibiotics.

[59] Herzog K., Engeler Dusel J., Hugentobler M., Beutin L., Sägesser G., Stephan R., Hächler H., Nüesch-Inderbinen M. (2014). Diarrheagenic Enteroaggregative *Escherichia coli* Causing Urinary Tract Infection and Bacteremia Leading to Sepsis. Infection.

[60] Mandomando I., Vubil D., Boisen N., Quintó L., Ruiz J., Sigaúque B., Nhampossa T., Garrine M., Massora S., Aide P. (2020). *Escherichia coli* ST131 Clones Harbouring AggR and AAF/V Fimbriae Causing Bacteremia in Mozambican Children: Emergence of New Variant of FimH27 Subclone. PLoS Negl. Trop. Dis..

[61] Paramita R.I., Nelwan E.J., Fadilah F., Renesteen E., Puspandari N., Erlina L. (2021). Genome-Based Characterization of *Escherichia coli* Causing Bloodstream Infection through next-Generation Sequencing. PLoS ONE.

[62] Riveros M., García W., García C., Durand D., Mercado E., Ruiz J., Ochoa T.J. (2017). Molecular and Phenotypic Characterization of Diarrheagenic *Escherichia coli* Strains Isolated from Bacteremic Children. Am. J. Trop. Med. Hyg..

[63] Moraes C.T.P., Longo J., Silva L.B., Pimenta D.C., Carvalho E., Morone M.S.L.C., da Rós N., Serrano S.M.T., Santos A.C.M., Piazza R.M.F. (2020). Surface Protein Dispersin of Enteroaggregative *Escherichia coli* Binds Plasminogen That Is Converted Into Active Plasmin. Front. Microbiol..

[64] Chaudhuri R.R., Sebaihia M., Hobman J.L., Webber M.A., Leyton D.L., Goldberg M.D., Cunningham A.F., Scott-Tucker A., Ferguson P.R., Thomas C.M. (2010). Complete Genome Sequence and Comparative Metabolic Profiling of the Prototypical Enteroaggregative *Escherichia coli* Strain 042. PLoS ONE.

[65] Alav I., Sutton J.M., Rahman K.M. (2018). Role of Bacterial Efflux Pumps in Biofilm Formation. J. Antimicrob. Chemother..

[66] Bay D.C., Stremick C.A., Slipski C.J., Turner R.J. (2017). Secondary Multidrug Efflux Pump Mutants Alter *Escherichia coli* Biofilm Growth in the Presence of Cationic Antimicrobial Compounds. Res. Microbiol..

[67] Johnson J.R., Gajewski A., Lesse A.J., Russo T.A. (2003). Extraintestinal Pathogenic *Escherichia coli* as a Cause of Invasive Nonurinary Infections. J. Clin. Microbiol..

[68] Petro C.D., Duncan J.K., Seldina Y.I., Allué-Guardia A., Eppinger M., Riddle M.S., Tribble D.R., Johnson R.C., Dalgard C.L., Sukumar G. (2020). Genetic and Virulence Profiles of Enteroaggregative *Escherichia coli* (EAEC) Isolated From Deployed Military Personnel (DMP) With Travelers’ Diarrhea. Front. Cell. Infect. Microbiol..

[69] Spurbeck R.R., Dinh P.C., Walk S.T., Stapleton A.E., Hooton T.M., Nolan L.K., Kim K.S., Johnson J.R., Mobley H.L.T. (2012). *Escherichia coli* Isolates That Carry Vat, Fyua, Chua, and Yfcv Efficiently Colonize the Urinary Tract. Infect. Immun..

[70] Flament-simon S.C., de Toro M., García V., Blanco J.E., Blanco M., Alonso M.P., Goicoa A., Díaz-gonzález J., Nicolas-chanoine M.H., Blanco J. (2020). Molecular Characteristics of Extraintestinal Pathogenic *E. coli* (Expec), Uropathogenic *E. coli* (Upec), and Multidrug Resistant *E. coli* Isolated from Healthy Dogs in Spain. Whole Genome Sequencing of Canine St372 Isolates and Comparison with Human Isolates Causing Extraintestinal Infections. Microorganisms.

[71] Nascimento J.A.S., Santos F.F., Valiatti T.B., Santos-Neto J.F., Santos A.C.M., Cayô R., Gales A.C., Gomes T.A.T. (2021). Frequency and Diversity of Hybrid *Escherichia coli* Strains Isolated from Urinary Tract Infections. Microorganisms.

[72] Santos A.C.M., Santos F.F., Silva R.M., Gomes T.A.T. (2020). Diversity of Hybrid- and Hetero-Pathogenic *Escherichia coli* and Their Potential Implication in More Severe Diseases. Front. Cell. Infect. Microbiol..

[73] Santos A.C.M., Zidko A.C.M., Pignatari A.C., Silva R.M. (2013). Assessing the Diversity of the Virulence Potential of *Escherichia coli* Isolated from Bacteremia in São Paulo, Brazil. Braz. J. Med. Biol. Res..

[74] Bhargava S., Johnson B.B., Hwang J., Harris T.A., George A.S., Muir A., Dorff J., Okeke I.N. (2009). Heat-Resistant Agglutinin 1 Is an Accessory Enteroaggregative *Escherichia coli* Colonization Factor. J. Bacteriol..

[75] Blackburn D., Husband A., Saldaña Z., Nada R.A., Klena J., Qadri F., Girón J.A. (2009). Distribution of the *Escherichia coli* Common Pilus among Diverse Strains of Human Enterotoxigenic *E. coli*. J. Clin. Microbiol..

[76] Mancini J., Weckselblatt B., Chung Y.K., Durante J.C., Andelman S., Glaubman J., Dorff J.D., Bhargava S., Lijek R.S., Unger K.P. (2011). The Heat-Resistant Agglutinin Family Includes a Novel Adhesin from Enteroaggregative *Escherichia coli* Strain 60A. J. Bacteriol..

[77] Rendón M.A., Saldaña Z., Erdem A.L., Monteiro-Neto V., Vázquez A., Kaper J.B., Puente J.L., Girón J.A. (2007). Commensal and Pathogenic *Escherichia coli* Use a Common Pilus Adherence Factor for Epithelial Cell Colonization. Proc. Natl. Acad. Sci. USA.

[78] Avelino F., Saldaña Z., Islam S., Monteiro-Neto V., Dall’Agnol M., Eslava C.A., Girón J.A. (2010). The majority of Enteroaggregative *Escherichia coli* Strains Produce the *E. coli* Common Pilus when Adhering to Cultured Epithelial Cells. Int. J. Med. Microbiol..

[79] Abreu A.G., Bueris V., Porangaba T.M., Sircili M.P., Navarro-Garcia F., Elias W.P. (2013). Autotransporter Protein-Encoding Genes of Diarrheagenic *Escherichia coli* Are Found in Both Typical and Atypical Enteropathogenic *E. coli* Strains. Appl. Environ. Microbiol..

[80] Restieri C., Garriss G., Locas M.C., Dozois C.M. (2007). Autotransporter-Encoding Sequences Are Phylogenetically Distributed among *Escherichia coli* Clinical Isolates and Reference Strains. Appl. Environ. Microbiol..

[81] Tapader R., Chatterjee S., Singh A.K., Dayma P., Haldar S., Pal A., Basu S. (2014). The High Prevalence of Serine Protease Autotransporters of Enterobacteriaceae (SPATEs) in *Escherichia coli* Causing Neonatal Septicemia. Eur. J. Clin. Microbiol. Infect. Dis..

[82] Flores-Sanchez F., Chavez-Dueñas L., Sanchez-Villamil J., Navarro-Garcia F. (2020). Pic Protein from Enteroaggregative *E. coli* Induces Different Mechanisms for Its Dual Activity as a Mucus Secretagogue and a Mucinase. Front. Immunol..

[83] Guyer D.M., Radulovic S., Jones F.E., Mobley H.L.T. (2002). Sat, the Secreted Autotransporter Toxin of Uropathogenic *Escherichia coli*, Is a Vacuolating Cytotoxin for Bladder and Kidney Epithelial Cells. Infect. Immun..

[84] Nava-Acosta R., Navarro-Garcia F. (2013). Cytokeratin 8 Is an Epithelial Cell Receptor for Pet, a Cytotoxic Serine Protease Autotransporter of Enterobacteriaceae. mBio.

[85] Maldonado-Contreras A., Birtley J.R., Boll E., Zhao Y., Mumy K.L., Toscano J., Ayehunie S., Reinecker H.C., Stern L.J., McCormick B.A. (2017). Shigella Depends on SepA to Destabilize the Intestinal Epithelial Integrity via Cofilin Activation. Gut Microbes.

[86] Vieira P.C.G., Espinoza-Culupú A.O., Nepomuceno R., Alves M.R., Lebrun I., Elias W.P., Ruiz R.C. (2020). Secreted Autotransporter Toxin (Sat) Induces Cell Damage during Enteroaggregative *Escherichia coli* Infection. PLoS ONE.

[87] Abreu A.G., Abe C.M., Nunes K.O., Moraes C.T., Chavez-Dueñas L., Navarro-Garcia F., Barbosa A.S., Piazza R.M., Elias W.P. (2016). The Serine Protease Pic as a Virulence Factor of Atypical Enteropathogenic *Escherichia coli*. Gut Microbes.

[88] Correa G.B., Freire C.A., Dibo M., Huerta-Cantillo J., Navarro-Garcia F., Barbosa A.S., Elias W.P., Moraes C.T.P. (2024). Plasmid-Encoded Toxin of *Escherichia coli* Cleaves Complement System Proteins and Inhibits Complement-Mediated Lysis in Vitro. Front. Cell. Infect. Microbiol..

[89] Orth D., Ehrlenbach S., Brockmeyer J., Khan A.B., Huber G., Karch H., Sarg B., Lindner H., Würzner R. (2010). EspP, a Serine Protease of Enterohemorrhagic *Escherichia coli*, Impairs Complement Activation by Cleaving Complement Factors C3/C3b and C5. Infect. Immun..

[90] Herzer P.J., Inouye S., Inouye M., Whittam T.S. (1990). Phylogenetic Distribution of Branched RNA-Linked Multicopy Single-Stranded DNA among Natural Isolates of *Escherichia coli*. J. Bacteriol..

[91] LeCointre G., Rachdi L., Darlu P., Denamur E. (1998). *Escherichia coli* Molecular Phylogeny Using the Incongruence Length Difference Test. Mol. Biol. Evol..

[92] Okeke I.N., Wallace-Gadsden F., Simons H.R., Matthews N., Labar A.S., Hwang J., Wain J. (2010). Multi-Locus Sequence Typing of Enteroaggregative *Escherichia coli* Isolates from Nigerian Children Uncovers Multiple Lineages. PLoS ONE.

[93] Scheutz F., Nielsen E.M., Frimodt-Møller J., Boisen N., Morabito S., Tozzoli R., Nataro J.P., Caprioli A. (2011). Characteristics of the Enteroaggregative Shiga Toxin/Verotoxin-Producing *Escherichia coli* O104:H4 Strain Causing the Outbreak of Haemolytic Uraemic Syndrome in Germany, May to June 2011. Eurosurveillance.

[94] Mariani-Kurkdjian P., Lemâitre C., Bidet P., Perez D., Boggini L., Kwon T., Bonacorsi S. (2014). Haemolytic-Uraemic Syndrome with Bacteraemia Caused by a New Hybrid *Escherichia coli* Pathotype. New Microbes New Infect..

[95] Kessler R., Nisa S., Hazen T.H., Horneman A., Amoroso A., Rasko D.A., Donnenberg M.S. (2015). Diarrhea, Bacteremia and Multiorgan Dysfunction Due to an Extraintestinal Pathogenic *Escherichia coli* Strain with Enteropathogenic *E. coli* Genes. Pathog. Dis..

[96] Benjelloun-Touimi Z., Sansonetti P.J., Parsot C. (1995). SepA, the Major Extracellular Protein of *Shigella flexneri*: Autonomous Secretion and Involvement in Tissue Invasion. Mol. Microbiol..

[97] Sambrook J., Fritsch E.F., Maniatis T. (1989). Molecular Cloning: A Laboratory Manual.

[98] Taddei C.R., Fasano A., Ferreira A.J.P., Trabulsi L.R., Martinez M.B. (2005). Secreted Autotransporter Toxin Produced by a Diffusely Adhering *Escherichia coli* Strain Causes Intestinal Damage in Animal Model Assays. FEMS Microbiol. Lett..

[99] Chen S., Zhou Y., Chen Y., Gu J. (2018). Fastp: An Ultra-Fast All-in-One FASTQ Preprocessor. Bioinformatics.

[100] Schubert M., Lindgreen S., Orlando L. (2016). AdapterRemoval v2: Rapid Adapter Trimming, Identification, and Read Merging. BMC Res. Notes.

[101] Langmead B., Salzberg S.L. (2012). Fast Gapped-Read Alignment with Bowtie 2. Nat. Methods.

[102] Bankevich A., Nurk S., Antipov D., Gurevich A.A., Dvorkin M., Kulikov A.S., Lesin V.M., Nikolenko S.I., Pham S., Prjibelski A.D. (2012). SPAdes: A New Genome Assembly Algorithm and Its Applications to Single-Cell Sequencing. J. Comput. Biol..

[103] Gurevich A., Saveliev V., Vyahhi N., Tesler G. (2013). QUAST: Quality Assessment Tool for Genome Assemblies. Bioinformatics.

[104] Beghain J., Bridier-Nahmias A., Le Nagard H., Denamur E., Clermont O. (2018). ClermonTyping: An Easy-to-Use and Accurate in Silico Method for Escherichia Genus Strain Phylotyping. Microb. Genom..

[105] Hall B.G., Nisbet J. (2023). Building Phylogenetic Trees from Genome Sequences with KSNP4. Mol. Biol. Evol..

[106] Price M.N., Dehal P.S., Arkin A.P. (2010). FastTree 2—Approximately Maximum-Likelihood Trees for Large Alignments. PLoS ONE.

[107] Altschul S.F., Gish W., Miller W., Myers E.W., Lipman D.J. (1990). Basic Local Alignment Search Tool. J. Mol. Biol..

[108] Ihaka R., Gentleman R. (1996). R: A Language for Data Analysis and Graphics. J. Comput. Graph. Stat..

[109] Sullivan M.J., Petty N.K., Beatson S.A. (2011). Easyfig: A Genome Comparison Visualizer. Bioinformatics.

[110] Koontz L. (2014). TCA Precipitation. Methods in Enzymology.

[111] Laemmli U.K. (1970). Cleavage of Structural Proteins during the Assembly of the Head of Bacteriophage T4. Nature.

[112] Chevallet M., Luche S., Rabilloud T. (2006). Silver Staining of Proteins in Polyacrylamide Gels. Nat. Protoc..

[113] Vilhena-Costa A.B., Piazza R.M., Nara J.M., Trabulsi L.R., Martinez M.B. (2006). Slot Blot Immunoassay as a Tool for Plasmid-Encoded Toxin Detection in Enteroaggregative *Escherichia coli* Culture Supernatants. Diagn. Microbiol. Infect. Dis..

[114] Navarro-Garcia F., Gutierrez-Jimenez J., Garcia-Tovar C., Castro L.A., Salazar-Gonzalez H., Cordova V. (2010). Pic, an Autotransporter Protein Secreted by Different Pathogens in the Enterobacteriaceae Family, is a Potent Mucus Secretagogue. Infect. Immun..

[115] Miajlovic H., Aogáin M.M., Collins C.J., Rogers T.R., Smith S.G.J. (2016). Characterization of *Escherichia coli* Bloodstream Isolates Associated with Mortality. J. Med. Microbiol..

