# Supplementary material for: Genomic Dissection of an Enteroaggregative Escherichia coli Strain Isolated from Bacteremia Reveals Insights into Its Hybrid Pathogenic Potential
Source: Int J Mol Sci. 2024 Aug 26;25(17):9238. doi: 10.3390/ijms25179238 (PMC11394720; doi:10.3390/ijms25179238)
Supplement: Supplementary file 1 [file ijms-25-09238-s001.zip › Fig. S1.pdf]

Fig. S1. Alignment between the predicted amino acid sequences of the Pet protein from EC092 and EAEC 042 strains.

```
EC092     MNKIYSIKYSAATGGLIAVSELAKKVTCKTNRKISAALLSLAVISYTNIIYAANMDISKV   60
042       MNKIYSIKYSAATGGLIAVSELAKKVICKTNRKISAALLSLAVISYTNIIYAANMDISKA   60
          ************************** ********************************.

EC092     WARDYLDLAQNKGVFQPGSTHVKIKLKDGTDFSFPALPVPDFSSATANGAATSIGGAYAV   120
042       WARDYLDLAQNKGVFQPGSTHVKIKLKDGTDFSFPALPVPDFSSATANGAATSIGGAYAV   120
          ************************************************************

             124                          153
EC092     TVAHNAKNKSSANYQTYGSTQYTQINRMTTGNDFSIQRLNKYVVETRGADTSFNYNENNQ   180
042       TVAHNAKNKSSANYQTYGSTQYTQINRMTTGNDFSIQRLNKYVVETRGADTSFNYNENNQ   180
          ************************************************************

EC092     NIIDRYGVDVGNGKKEIIGFRVGSGNTTFSGIKTSQTYQADLLSASLFHITNLRANTVGG   240
042       NIIDRYGVDVGNGKKEIIGFRVGSGNTTFSGIKTSQTYQADLLSASLFHITNLRANTVGG   240
          ************************************************************

                            260
EC092     NKVEYENDSYFTNLTTNGDSGSGVYVFDHKEDKWVLLGTTHGIIGNGKTQKTYVTPFDSK   300
042       NKVEYENDSYFTNLTTNGDSGSGVYVFDNKEDKWVLLGTTHGIIGNGKTQKTYVTPFDSK   300
          ****************************:*******************************

EC092     TTNELKQLFIQNVNIDNNTATIGGGKITIGNTTQDIEKNKNDQNKDLVFSGGGKISLKEN   360
042       TTNELKQLFIQNVNIDNNTATIGGGKITIGNTTQDIEKNKNNQNKDLVFSGGGKISLKEN   360
          *****************************************:******************

EC092     LDLGYGGFIFDENKKYTVSAEGNNNVTFKGAGIDIGKGSTVDWNIKYASNDALHKIGEGS   420
042       LDLGYGGFIFDENKKYTVSAEGNNNVTFKGAGIDIGKGSTVDWNIKYASNDALHKIGEGS   420
          ************************************************************

EC092     LNVIQAQNTNLKTGNGTVILGAQKTFNNIYVAGGPGTVQLNAENALGEGDYAGIFFTENG   480
042       LNVIQAQNTNLKTGNGTVILGAQKTFNNIYVAGGPGTVQLNAENALGEGDYAGIFFTENG   480
          ************************************************************

EC092     GKLDLNGHNQTFKKIAATDSGTTITNSNTTKESVLSVNNQNNYIYHGNVDGNVRLEHHLD   540
042       GKLDLNGHNQTFKKIAATDSGTTITNSNTTKESVLSVNNQNNYIYHGNVDGNVRLEHHLD   540
          ************************************************************

EC092     TKQNNARLILDGDIQANSISIKNAPLVMQGHATDHAIFRTTKTNNCPEFLCGVDWVTRIK   600
042       TKQDNARLILDGDIQANSISIKNAPLVMQGHATDHAIFRTTKTNNCPEFLCGVDWVTRIK   600
```

```
              ***:********************************************************

EC092         NAENSVNQKNKTTYKSNNQVSDLSQPDWETRKFRFDNLNIEDSSLSIARNADVEGNIQAK      660
042           NAENSVNQKNKTTYKSNNQVSDLSQPDWETRKFRFDNLNIEDSSLSIARNADVEGNIQAK      660
              ************************************************************

EC092         NSVINIGDKTAYIDLYSGKNITGAGFAFRQDIKSGDSIGESKFTGGIMATDGSISIGDKA      720
042           NSVINIGDKTAYIDLYSGKNITGAGFTFRQDIKSGDSIGESKFTGGIMATDGSISIGDKA      720
              **************************:*********************************

EC092         IVTLNTVSSLDRTALTIHKGANVTASSSLFTTSNIKSGGDLTLTGATESTGEITPSMFYA      780
042           IVTLNTVSSLDRTALTIHKGANVTASSSLFTTSNIKSGGDLTLTGATESTGEITPSMFYA      780
              ************************************************************

EC092         AGGYELTEDGANFTAKNQASVTGDIKSEKAAKLSFGSADKDNSATSYSQFALAMLDGFDT      840
042           AGGYELTEDGANFTAKNQASVTGDIKSEKAAKLSFGSADKDNSATRYSQFALAMLDGFDT      840
              ********************************************* **************

EC092         SYQGSIKAAQSSLAMNNALWKVTGNSELKKLNSTGSMVLFNGGKNIFNTLTVDELTTSNS      900
042           SYQGSIKAAQSSLAMNNALWKVTGNSELKKLNSTGSMVLFNGGKNIFNTLTVDELTTSNS      900
              ************************************************************

EC092         AFVMRTNTQQADQLIVKNKLEGANNLLLVDFIEKKGNDKNGLNIDLVKAPENTSKDVFKT      960
042           AFVMRTNTQQADQLIVKNKLEGANNLLLVDFIEKKGNDKNGLNIDLVKAPENTSKDVFKT      960
              ************************************************************

EC092         ETQTIGFSDVTPEIKQQEKDGKSVWTLTGYKTVANADAAKKATSLMSGGYKAFLAEVNNL      1020
042           ETQTIGFSDVTPEIKQQEKDGKSVWTLTGYKTVANADAAKKATSLMSGGYKAFLAEVNNL      1020
              ************************************************************

EC092         NKRMGDLRDINGEAGAWARIMSGTGSAGGGFSDNYTHVQVGADNKHELDGLDLFTGVTMT      1080
042           NKRMGDLRDINGEAGAWARIMSGTGSAGGGFSDNYTHVQVGADNKHELDGLDLFTGVTMT      1080
              ************************************************************

EC092         YTDSHAGSDAFSGETKSVGAGLYASAMFESGAYIDLIGKYVHHDNEYTATFAGLGTRDYS      1140
042           YTDSHAGSDAFSGETKSVGAGLYASAMFESGAYIDLIGKYVHHDNEYTATFAGLGTRDYS      1140
              ************************************************************

EC092         SHSWYAGAEVGYRYHVTDSAWIEPQAELVYGAVSGKQFSWKDQGMNLTMKDKDFNPLIGR      1200
042           SHSWYAGAEVGYRYHVTDSAWIEPQAELVYGAVSGKQFSWKDQGMNLTMKDKDFNPLIGR      1200
              ************************************************************
```

```
EC092       TGVDVGKSFSGKDWKVTARAGLGYQFDLFANGETVLRDASGEKRIKGEKDGRMLMNVGLN     1260
042         TGVDVGKSFSGKDWKVTARAGLGYQFDLFANGETVLRDASGEKRIKGEKDGRMLMNVGLN     1260
            ************************************************************

EC092       AEIRDNVRFGLEFEKSAFGKYNVDNAINANFRYSF     1295
042         AEIRDNVRFGLEFEKSAFGKYNVDNAINANFRYSF     1295
            ***********************************
```

Alignment was performed on the virtual platform of Cluster Omega and seven amino acid changes were identified (highlighted in orange color). Structural main features are preserved and highlighted as follows: the intact serine protease motif, GDSGS (yellow); the catalytic triad; His124, Asp153 and Ser260 (green) and the linker domain site (light blue).
