# Supplementary material for: Genomic Dissection of an Enteroaggregative Escherichia coli Strain Isolated from Bacteremia Reveals Insights into Its Hybrid Pathogenic Potential
Source: Int J Mol Sci. 2024 Aug 26;25(17):9238. doi: 10.3390/ijms25179238 (PMC11394720; doi:10.3390/ijms25179238)
Supplement: Supplementary file 1 [file ijms-25-09238-s001.zip › Fig. S10.pdf]

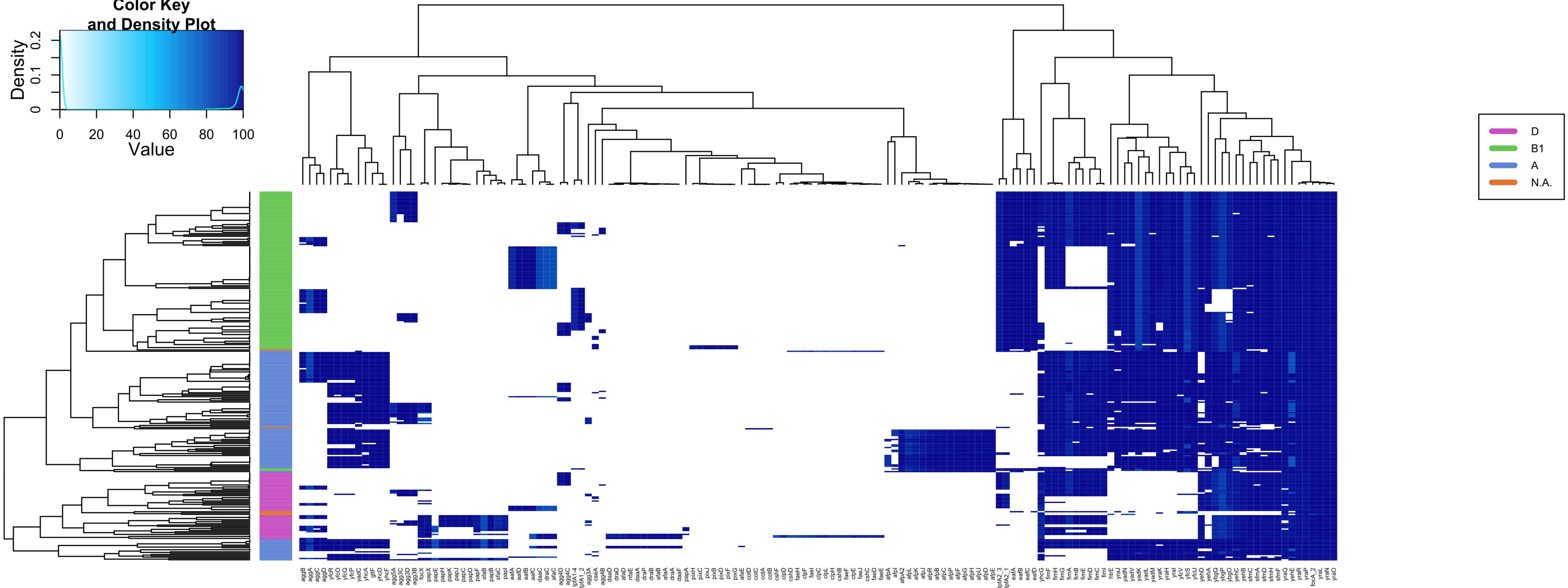

Fig. S10. Heatmap indicating presence or absence of fimbriae-encoding genes.

Each white and blue square indicates the presence or absence of a gene associated with virulence. Strain names are color-coded for phylogroup membership. EC092 is shown in red. Also shown are the cluster dendrograms representing t he hierarchical clustering of the genomes (left) and genes (top) based on the presence/absence data.
