# Supplementary material for: Genomic Dissection of an Enteroaggregative Escherichia coli Strain Isolated from Bacteremia Reveals Insights into Its Hybrid Pathogenic Potential
Source: Int J Mol Sci. 2024 Aug 26;25(17):9238. doi: 10.3390/ijms25179238 (PMC11394720; doi:10.3390/ijms25179238)
Supplement: Supplementary file 1 [file ijms-25-09238-s001.zip › Fig. S15.pdf]

Fig. S15. Alignment of blocks of the genome from strains EC092, K44V1, and K45V1.

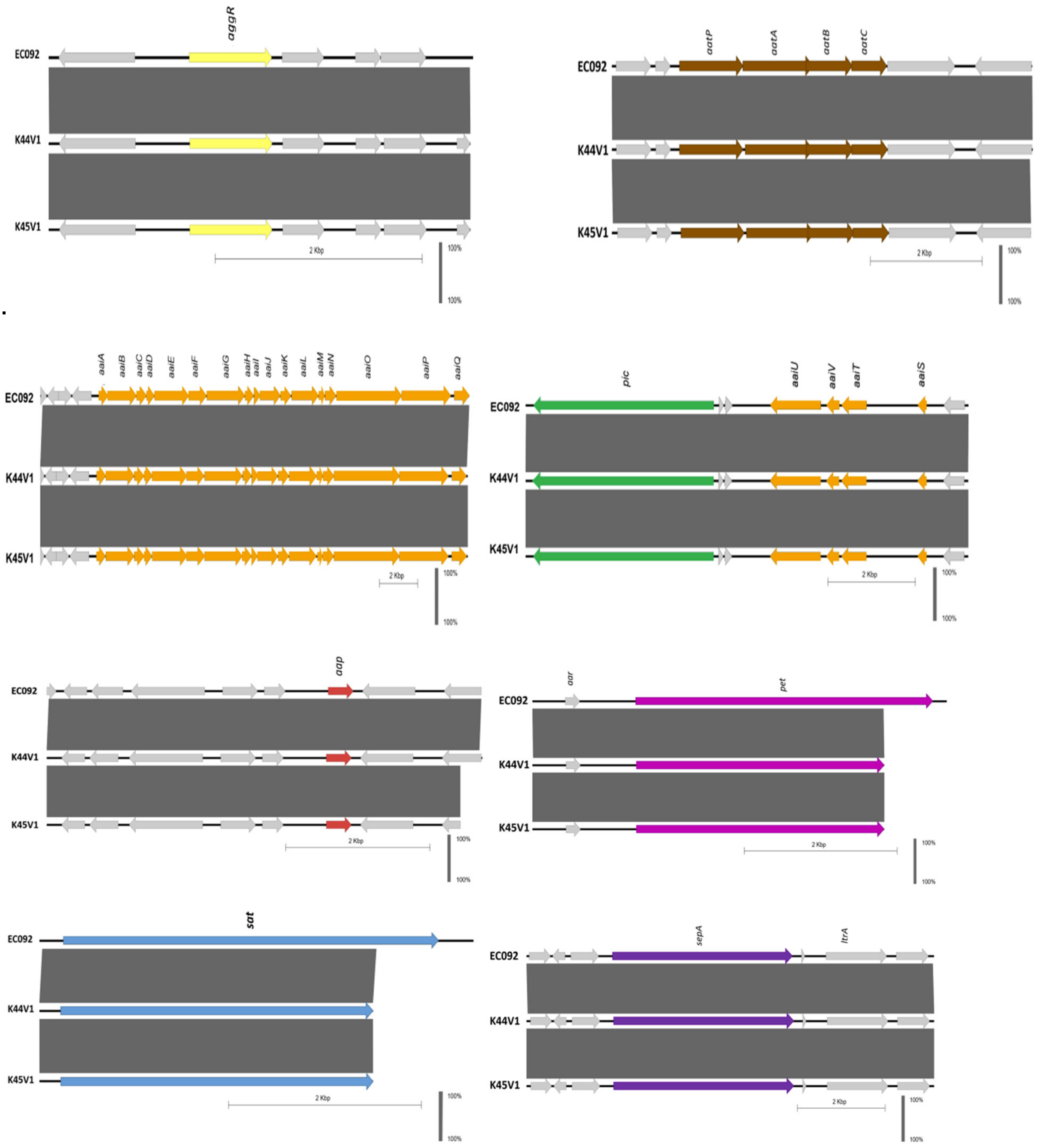

These figures depict the neighborhoods of genes and operons, providing insights into their genomic context. Each genome is represented by a solid black line and each gene is represented by an arrow; the gray columns between genomes indicate the level of similarity, according to the provided key; a scale bar is also shown. Each figure represents a specific gene or operon, with

neighboring genes displayed in gray. The gene products of interest are highlighted with distinct colors: *aggR* in yellow, *aat*A-P operon in brown, *aai*A-Q operon in orange, pic gene and *aai*S-U operon in green and orange respectively, *aap* gene in red, *pet* gene in lilac, *sat* gene in blue, and *sep*A gene in purple. These visualizations contribute to the understanding of the genetic relationships and context of these elements in the genomes of the studied strains, highlighting potential interactions and associations as well as giving a robust support to the orthology of these genes.
