# Supplementary material for: Genomic Dissection of an Enteroaggregative Escherichia coli Strain Isolated from Bacteremia Reveals Insights into Its Hybrid Pathogenic Potential
Source: Int J Mol Sci. 2024 Aug 26;25(17):9238. doi: 10.3390/ijms25179238 (PMC11394720; doi:10.3390/ijms25179238)
Supplement: Supplementary file 1 [file ijms-25-09238-s001.zip › Fig. S16.pdf]

Fig. S16. Plasmid content of the EC092 strain

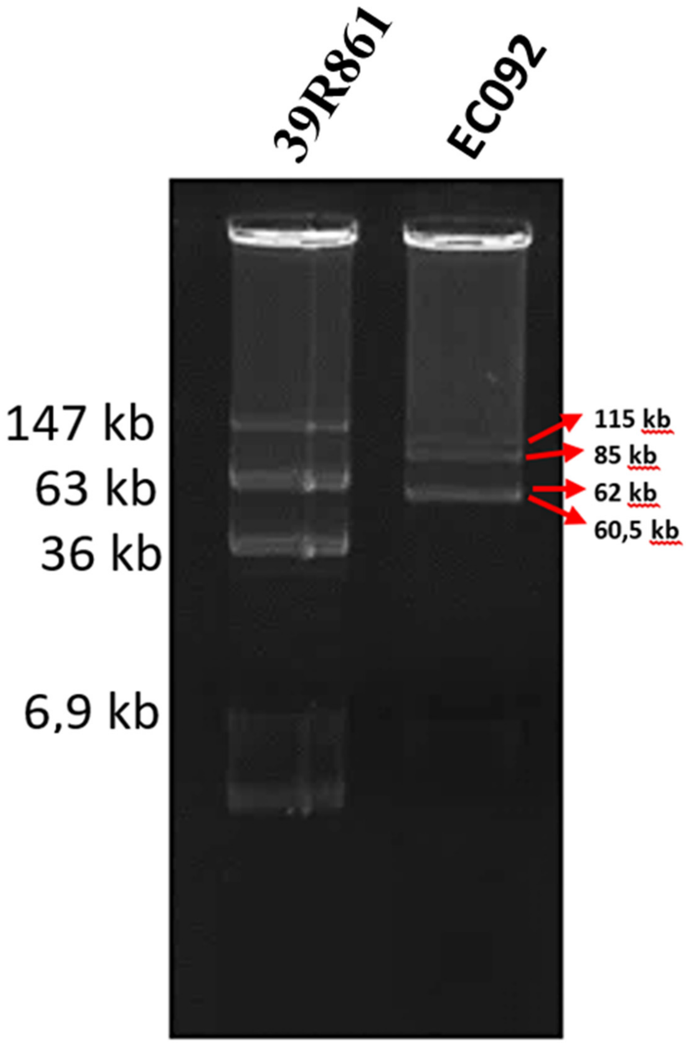

Plasmids were obtained by alkaline extraction, followed by electrophoresis in 0.8% agarose gel in Tris-Borate-EDTA (TBE) buffer. Approximate sizes were predicted based on the plasmid migration in agarose gel. The *E. coli* strain 39R861 represents the standard strain containing plasmids of known molecular weights (BIMBOIM, H. C.; DOLY, J. A rapid alkaline extraction procedure for screening recombinant plasmid DNA. Nucleic. Acids Res., v. 7, p. 1513-1523, 1979).
