# Supplementary material for: Genomic Dissection of an Enteroaggregative Escherichia coli Strain Isolated from Bacteremia Reveals Insights into Its Hybrid Pathogenic Potential
Source: Int J Mol Sci. 2024 Aug 26;25(17):9238. doi: 10.3390/ijms25179238 (PMC11394720; doi:10.3390/ijms25179238)
Supplement: Supplementary file 1 [file ijms-25-09238-s001.zip › Fig. S2.pdf]

Fig. S2. Alignment between the predicted amino acid sequences of the Pic protein of strains EC092 and EAEC 042.

```
EC092       MNKVYSLKYCPVTGGLIAVSELARRVIKKTCRRLTHILLAGIPAICLCYSQISQAGIVRS    60
042         MNKVYSLKYCPVTGGLIAVSELARRVIKKTCRRLTHILLAGIPAICLCYSQISQAGIVRS    60
            ************************************************************

EC092       DIAYQIYRDFAENKGLFVPGANDIPVYDKDGKLVGRLGKAPMADFSSVSSNGVATLVSPQ    120
042         DIAYQIYRDFAENKGLFVPGANDIPVYDKDGKLVGRLGKAPMADFSSVSSNGVATLVSPQ    120
            ************************************************************

                  127                         155
EC092       YIVSVKHNGGYRSVSFGNGKNTYSLVDRNNHPSIDFHAPRLNKLVTEVIPSAVTSEGTKA    180
042         YIVSVKHNGGYRSVSFGNGKNTYSLVDRNNHPSIDFHAPRLNKLVTEVIPSAVTSEGTKA    180
            ************************************************************

EC092       NAYKYTERYTAFYRVGSGTQYTKDKDGNLVKVAGGYAFKTGGTTGVPLISDATIVSNPGQ    240
042         NAYKYTERYTAFYRVGSGTQYTKDKDGNLVKVAGGYAFKTGGTTGVPLISDATIVSNPGQ    240
            ************************************************************

                             258
EC092       TYNPVNGPLPDYGAPGDSGSPLFAYDKQQKKWVIVAVLRAYAGINGATNWWNVIPTDYLN    300
042         TYNPVNGPLPDYGAPGDSGSPLFAYDKQQKKWVIVAVLRAYAGINGATNWWNVIPTDYLN    300
            ************************************************************

EC092       QVMQDDFDAPVDFVSGLGPLNWTYDKTSGTGTLSQGSKNWTMHGQKDNDLNAGKNLVFSG    360
042         QVMQDDFDAPVDFVSGLGPLNWTYDKTSGTGTLSQGSKNWTMHGQKDNDLNAGENLVFSG    360
            *****************************************************:******

EC092       QNGAIILKDSVTQGAGYLEFKDSYTVSAESGKTWTGAGIITDKGTNVTWKVNGVAGDNLH    420
042         QNGAIILKDSVTQGAGYLEFKDSYPVSAESGKTWTGAGIITDKGTNVTWKVNGVAGDNLH    420
            ************************ ***********************************

EC092       KLGEGTLTINGTGVNPGGLKTGDGIVVLNQQADTAGNIQAFSSVNLASGRPTVVLGDARQ    480
042         KLGEGTLTINGTGVNPGGLKTGDGIVVLNQQADTAGNIQAFSSVNLASGRPTVVLGDARQ    480
            ************************************************************

EC092       VNPDNISWGYRGGKLDLNGNAVTFTRLQAADYGAVITNNAQQKSQLLLDLKAQDTNVSEP    540
042         VNPDNISWGYRGGKLDLNGNAVTFTRLQAADYGAVITNNAQQKSQLLLDLKAQDTNVSEP    540
            ************************************************************

EC092       TIGNISPFGGTGTPGNLYSMILNSQTRFYILKSASYGNTLWGNSLNDPAQWEFVGMDKNK    600
042         TIGNISPFGGTGTPGNLYSMILNSQTRFYILKSASYGNTLWGNSLNDPAQWEFVGMNKNK    600
            ********************************************************:***
```

```
EC092   AVQTVKDRILAGRAKQPVIFHGQLTGNMDVAIPQVPGGRKVIFDGSVNLPEGTLSQDSGT   660
042     AVQTVKDRILAGRAKQPVIFHGQLTGNMDVAIPQVPGGRKVIFDGSVNLPEGTLSQDSGT   660
        ************************************************************

EC092   LIFQGHPVIHASISGSAPVSLNQKDWENRQFTMKTLSLKDADFHLSRNASLNSDIKSDNS   720
042     LIFQGHPVIHASISGSAPVSLNQKDWENRQFTMKTLSLKDADFHLSRNASLNSDIKSDNS   720
        ************************************************************

EC092   HITLGSDRAFVDKNDGTGNYVIPEEGTSVPDTVNDRSQYEGNITLNHNSALDIGSRFTGG   780
042     HITLGSDRAFVDKNDGTGNYVIPEEGTSVPDTVNDRSQYEGNITLNHNSALDIGSRFTGG   780
        ************************************************************

EC092   IDAYDSAVSITSPDVLLTAPGAFAGSSLTVHDGGHLTALNGLFSDGHIQAGKNGKITLSG   840
042     IDAYDSAVSITSPDVLLTAPGAFAGSSLTVHDGGHLTALNGLFSDGHIQAGKNGKITLSG   840
        ************************************************************

EC092   TPVKDTANQYAPAVYLTDGYDLTGDNAALEITRGAHASGDIHASAASTVTIGSDTPAELA   900
042     TPVKDTANQYAPAVYLTDGYDLTGDNAALEITRGAHASGDIHASAASTVTIGSDTPAELA   900
        ************************************************************

EC092   SAETAASAFAGSLLEGYNAAFNGAITGGRADVSMHNALWTLGGDSAIHSLTVRNSRISSE   960
042     SAETAASAFAGSLLEGYNAAFNGAITGGRADVSMHNALWTLGGDSAIHSLTVRNSRISSE   960
        ************************************************************

EC092   GDRTFRTLTVNKLDATGSDFVLRTDLKNADKINVTEKATGSDNSLNVSFMNNPAQGQALN   1020
042     GDRTFRTLTVNKLDATGSDFVLRTDLKNADKINVTEKATGSDNSLNVSFMNNPAQGQALN   1020
        ************************************************************

EC092   IPLVTAPAGTSAEMFKAGTRVTGFSRVTPTLHVDTSGGNTKWILDGFKAEADKAAAAKAD   1080
042     IPLVTAPAGTSAEMFKAGTRVTGFSRVTPTLHVDTSGGNTKWILDGFKAEADKAAAAKAD   1080
        ************************************************************

EC092   SFMNAGYKNFMTEVNNLNKRMGDLRDTNGDAGAWARIMSGAGSADGGYSDNYTHVQVGFD   1140
042     SFMNAGYKNFMTEVNNLNKRMGDLRDTNGDAGAWARIMSGAGSADGGYSDNYTHVQVGFD   1140
        ************************************************************

EC092   KKHELDGVDLFTGVTMTYTDSSADSHAFSGKTKSVGGGLYASALFESGAYIDLIGKYIHH   1200
042     KKHELDGVDLFTGVTMTYTDSSADSHAFSGKTKSVGGGLYASALFESGAYIDLIGKYIHH   1200
        ************************************************************

EC092   DNDYTGNFASLGTKHYNTHSWYAGAETGYRYHLTEDTFIEPQAELVYGAVSGKTFRWKDG   1260
```

```
042         DNDYTGNFASLGTKHYNTHSWYAGAETGYRYHLTEDTFIEPQAELVYGAVSGKTFRWKDG     1260
            ************************************************************

EC092       DMDLSMKNRDFSPLVGRTGVELGKTFSGKDWSVTARAGTSWQFDLLNNGETVLRDASGEK     1320
042         DMDLSMKNRDFSPLVGRTGVELGKTFSGKDWSVTARAGTSWQFDLLNNGETVLRDASGEK     1320
            ************************************************************

EC092       RIKGEKDSRMLFNVGMNAQIKDNMRFGLEFEKSAFGKYNVDNAVNANFRYMF     1372
042         RIKGEKDSRMLFNVGMNAQIKDNMRFGLEFEKSAFGKYNVDNAVNANFRYMF     1372
            ****************************************************
```

Complete alignment between the amino acid sequence of the Pic protein of strain EC092 and prototype strain 042 (GenBank accession number: GCA_000027125.1). Alignment was performed on the Cluster Omega virtual platform and three amino acid changes (orange color) were identified. The intact serine protease motif (GDSGS) was located in both strains (yellow color). The catalytic triad (His127, Asp155 and Ser258) is marked in green and the conserved site of the linker domain in light blue.
