# Supplementary material for: Genomic Dissection of an Enteroaggregative Escherichia coli Strain Isolated from Bacteremia Reveals Insights into Its Hybrid Pathogenic Potential
Source: Int J Mol Sci. 2024 Aug 26;25(17):9238. doi: 10.3390/ijms25179238 (PMC11394720; doi:10.3390/ijms25179238)
Supplement: Supplementary file 1 [file ijms-25-09238-s001.zip › Fig. S3.pdf]

Fig. S3. Alignment between the predicted amino acid sequences of the Sat protein of EC092 and UPEC CFT073 strains.

```
EC092       MNKIYSLKYSAATGGLIAVSELAKRVSGKTNRKLVATMLSLAVAGTVNAANIDISNVWAR   60
CFT073      MNKIYSLKYSAATGGLIAVSELAKRVSGKTNRKLVATMLSLAVAGTVNAANIDISNVWAR   60
            ************************************************************

EC092       DYLDLAQNKGIFQPGATDVTITLKNGDKFSFHNLSIPDFSGAAASGAATAIGGSYSVTVA   120
CFT073      DYLDLAQNKGIFQPGATDVTITLKNGDKFSFHNLSIPDFSGAAASGAATAIGGSYSVTVA   120
            ************************************************************
           121                          149
EC092       HNKKNPQAAETQVYAQSSYKVVDRRNSNDFEIQRLNKFVVETVGATPAETNPTTYSDALE   180
CFT073      HNKKNPQAAETQVYAQSSYRVVDRRNSNDFEIQRLNKFVVETVGATPAETNPTTYSDALE   180
            *******************:****************************************

EC092       RYGIVTSDGSKKIIGFRAGSGGTSFINGESKISTNSAYSHDLLSASLFEVTQWDSYGMMI   240
CFT073      RYGIVTSDGSKKIIGFRAGSGGTSFINGESKISTNSAYSHDLLSASLFEVTQWDSYGMMI   240
            ************************************************************
                          256
EC092       YKNDKTFRNLEIFGDSGSGAYLYDNKLEKWVLVGTTHGIASVNGDQLTWITKYNDKLVSK   300
CFT073      YKNDKTFRNLEIFGDSGSGAYLYDNKLEKWVLVGTTHGIASVNGDQLTWITKYNDKLVSE   300
            ***********************************************************:

EC092       LKDTYSHKINLNGNNVTIKNTDITLHQNNADTTGTQEKITKDKDIVFTNGGNVLFKDNLD   360
CFT073      LKDTYSHKINLNGNNVTIKNTDITLHQNNADTTGTQEKITKDKDIVFTNGGDVLFKDNLD   360
            ***************************************************:********

EC092       FGSGGIIFDEGHEYNINGQGFTFKGAGIDIGKESIVNWNALYSSDDVLHKIGPGTLNVQK   420
CFT073      FGSGGIIFDEGHEYNINGQGFTFKGAGIDIGKESIVNWNALYSSDDVLHKIGPGTLNVQK   420
            ************************************************************

EC092       KQGANIKIGEGNVILNEEGTFNNIYLASGNGKVILNKDNSLGNDQYAGIFFTKRGGTLDL   480
CFT073      KQGANIKIGEGNVILNEEGTFNNIYLASGNGKVILNKDNSLGNDQYAGIFFTKRGGTLDL   480
            ************************************************************

EC092       NGHNQTFTRIAATDDGTTITNSDTTKEAVLAINNEDSYIYHGNINGNIKLTHNINSQDKK   540
CFT073      NGHNQTFTRIAATDDGTTITNSDTTKEAVLAINNEDSYIYHGNINGNIKLTHNINSQDKK   540
            ************************************************************

EC092       TNAKLILDGSVNTKNDVEVSNASLTMQGHATEHAIFRSTANHCSLVFLCGTDWVTVLKET   600
CFT073      TNAKLILDGSVNTKNDVEVSNASLTMQGHATEHAIFRSSANHCSLVFLCGTDWVTVLKET   600
            **************************************:*********************
```

```
EC092       ESSYNKKFNSDYKSNNQQTSFDQPDWKTGVFKFDTLHLNNADFSISRNANVEGNISANKS    660
CFT073      ESSYNKKFNSDYKSNNQQTSFDQPDWKTGVFKFDTLHLNNADFSISRNANVEGNISANKS    660
            ************************************************************

EC092       AITIGDKNAYIDNLAGKNITNNGFDFKQTISTNLSIGETKFTGGITAHNSQIAIGDQAVV    720
CFT073      AITIGDKNVYIDNLAGKNITNNGFDFKQTISTNLSIGETKFTGGITAHNSQIAIGDQAVV    720
            ********.***************************************************

EC092       TLNGATFLDNTPISIDKGAKVIAQNSMFTTKGIDISGELTMMGIPEQNSKAVTPGLHYAA    780
CFT073      TLNGATFLDNTPISIDKGAKVIAQNSMFTTKGIDISGELTMMGIPEQNSKTVTPGLHYAA    780
            **************************************************:*********

EC092       DGFRLSGGNANFIARNMASVTGNIYADDAATITLGQPETETPTISSAYQAWAETLLYGFD    840
CFT073      DGFRLSGGNANFIARNMASVTGNIYADDAATITLGQPETETPTISSAYQAWAETLLYGFD    840
            ************************************************************

EC092       TAYRGAITAPKATVSMNNAIWHLNSQSSINRLETKDSMVRFTGDNGKFTTLTVDNLTIDD    900
CFT073      TAYRGAITAPKATVSMNNAIWHLNSQSSINRLETKDSMVRFTGDNGKFTTLTVNNLTIDD    900
            *****************************************************:******

EC092       SAFVLRANLAQADQLVVNKSLSGKNNLLLVDFIEKNGNSNGLNIDLVSAPKGTAVDVFKA    960
CFT073      SAFVLRANLAQADQLVVNKSLSGKNNLLLVDFIEKNGNSNGLNIDLVSAPKGTAVDVFKA    960
            ************************************************************

EC092       TTRSIGFSDVTPVIEQKNDTDKATWTLIGYKSVANADAAKKATLLMSGGYKAFLAEVNNL    1020
CFT073      TTRSIGFSDVTPVIEQKNDTDKATWTLIGYKSVANADAAKKATLLMSGGYKAFLAEVNNL    1020
            ************************************************************

EC092       NKRMGDLRDINGESGAWARIMSGTGSAGGGFSDNYTHVQVGADNKHELDGLDLFTGVTMT    1080
CFT073      NKRMGDLRDINGESGAWARIISGTGSAGGGFSDNYTHVQVGADNKHELDGLDLFTGVTMT    1080
            ********************:***************************************

EC092       YTDSHAGSDAFSGETKSVGAGLYASAMFESGAYIDLIGKYVHHDNEYTATFAGLGTRDYS    1140
CFT073      YTDSHAGSDAFSGETKSVGAGLYASAMFESGAYIDLIGKYVHHDNEYTATFAGLGTRDYS    1140
            ************************************************************

EC092       SHSWYAGAEVGYRYHVTDSAWIEPQAELVYGAVSGKQFSWKDQGMNLTMKDKDFNPLIGR    1200
CFT073      SHSWYAGAEVGYRYHVTDSAWIEPQAELVYGAVSGKQFSWKDQGMNLTMKDKDFNPLIGR    1200
            ************************************************************

EC092       TGVDVGKSFSGKDWKVTARAGLGYQFDLFANGETVLRDASGEKRIKGEKDGRMLMNVGLN    1260
```

```
CFT073      TGVDVGKSFSGKDWKVTARAGLGYQFDLFANGETVLRDASGEKRIKGEKDGRMLMNVGLN     1260
            ************************************************************

EC092       AEIRDNLRFGLEFEKSAFGKYNVDNAINANFRYSF    1295
CFT073      AEIRDNLRFGLEFEKSAFGKYNVDNAINANFRYSF    1295
            ***********************************
```

Complete alignment between the amino acid sequence of the Sat protein of the EC092 strain and the UPEC prototype strain CFT073 (GenBank accession number: AAG30168.1). Alignment was performed on the Cluster Omega virtual platform and eight amino acid changes were identified (orange color). The intact serine protease motif (GDSGS) was located in both strains (yellow color). The catalytic triad (His121, Asp149 and Ser256) is marked in green and the conserved site of the linker domain in light blue.
