# Supplementary material for: Genomic Dissection of an Enteroaggregative Escherichia coli Strain Isolated from Bacteremia Reveals Insights into Its Hybrid Pathogenic Potential
Source: Int J Mol Sci. 2024 Aug 26;25(17):9238. doi: 10.3390/ijms25179238 (PMC11394720; doi:10.3390/ijms25179238)
Supplement: Supplementary file 1 [file ijms-25-09238-s001.zip › Fig. S4.pdf]

Fig. S4. Alignment between predicted amino acid sequences of SepA protein from EC092 and *Shigella flexneri* M90T strains.

```
EC092     MNKIYYLKYCHITKSLIAVSELARRVTCKSHRRLSRRVILTSVAALSLSSAWPALSATVS    60
M90T      MNKIYYLKYCHITKSLIAVSELARRVTCKSHRRLSRRVILTSVAALSLSSAWPALSATVS    60
          ************************************************************

EC092     AEIPYQIFRDFAENKGQFTPGTTNISIYDKQGNLVGKLDKAPMADFSSATITTGSLPPGN    120
M90T      AEIPYQIFRDFAENKGQFTPGTTNISIYDKQGNLVGKLDKAPMADFSSATITTGSLPPGD    120
          ***********************************************************:

                       134                         162
EC092     HTLYSPQYVVTAKHVSGSDTMSFGYAKNTYTAVGTNNNSGLDIKTRRLSKLVTEVAPAEV    180
M90T      HTLYSPQYVVTAKHVSGSDTMSFGYAKNTYTAVGTNNNSGLDIKTRRLSKLVTEVAPAEV    180
          ************************************************************

EC092     SDIGAVSGAYQAGGRFTAFYRLGGGMQYVKDKNGNRTQVYTNGGFLVGGTVSALNSYNNG    240
M90T      SDIGAVSGAYQAGGRFTEFYRLGGGMQYVKDKNGNRTQVYTNGGFLVGGTVSALNSYNNG    240
          ***************** ******************************************

                                     268
EC092     QMITAQTGDIF-NPANGPLANYLNMGDSGSPLFAYDSLQKKWVLIGVLSSGTNYGNNWVV    299
M90T      QMITAQTGDIFLIPPNGPLANYLNMGDSGSPLFAYDSLQKKWVLIGVLSSGTNYGNNWVV    300
          ***********  * *********************************************

EC092     TTQDFLGQQPQNDFDKTIAYTSGEGVLQWKYDAANGTGTLTQGNTTWDMHGKK-GNDLNA    358
M90T      TTQDFLGQQPQNDFDKTIAYTSGEGVLQWKYDAANGTGTLTQGNTTWGYAWKERKLILNA    360
          ***********************************************.   *:    ***

EC092     GKNLLFTGNNGEVVLQNSVNQGAGYLQFAGDYRVSALNGQTWMGGGIITDKGTHVLWQVN    418
M90T      GKNLLFTGNNGEVVLQNSVNQGAGYLQFAGDYRVSALNGQTWMGGGIITDKGTHVLWQVN    420
          ************************************************************

EC092     GVAGDNLHKTGEGTLTVNGTGVNAGGLKVGDGTVILNQQADADGKVQAFSSVGIASGRPT    478
M90T      GVAGDNLHKTGEGTLTVNGTGVNAGGLKVGDGTVILNQQADRDGKVQAFSSVGIASGRPT    480
          ***************************************** ******************

EC092     VVLSDSQQVNPDNISWGYRGGRLELNGNNLTFTRLQAADYGAIITNNSEKKSTVTLNLQT    538
M90T      VVLSDSQQVNPDNISWGYRGGRLELNGNNLTFTRLQAADYGAIITNNSEKKSTVTLDLQT    540
          ********************************************************:***

EC092     LKASDINVPVNTVSIFGGRGAPGDLYYDSSTKQYFILKASSYSPFFSDLNNSSVWQNVGK    598
M90T      LKASDINVPVNTVSIFGGRGAPGDLYYDSSTKQYFILKASSYSPFFSDLNNSSVWQNVGK    600
          ************************************************************
```

```
EC092     DRNKAIDTVKQQKIEASSQPYMYHGQLNGNMDVNIPQLSGKDVLALDGSVNLPEGSITKK     658
M90T      DHNKAIDTVKQQKIEASSQPYMYHGQLNGNMDVNIPQLSGKDVLALDGSVNLPEGSITKK     660
          *:**********************************************************

EC092     SGTLIFQGHPVIHAGTTTSSSQSDWETRQFTLEKLKLDAATFHLSRNGKMQGDINATNGS     718
M90T      SGTLIFQGHPVIHAGTTTSSSQSDWETRQFTLEKLKLDAATFHLSRNGKMQGDINATNGS     720
          ************************************************************

EC092     TVILGSSRVFTDRSDGTGNAVSSVEGSATATTVGDQSDYSGNVTLENKSSLQIMERFTGG     778
M90T      TVILGSSRVFTDRSDGTGNAVSSVEGSATATTVGDQSDYSGNVTLENKSSLQIMERFTGG     780
          ************************************************************

EC092     IEAYDSTVSVTSQNAVFDRVGSFVNSSLTLGKGAKLTAQSGIFSTGAVDVKENASLTLTG     838
M90T      IEAYDSTVSVTSQNAVFDRVGSFVNSSLTLGKGAKLTAQSGIFSTGAVDVKENASLTLTG     840
          ************************************************************

EC092     MPSAQKQGYYSPVISTTEGINLEDKASFSVKNMGYLSSDIHAGTTAATINLGDSDADAGK     898
M90T      MPSAQKQGYYSPVISTTEGINLEDNASFSVKNMGYLSSDIHAGTTAATINLGDSDADAGK     900
          ************************:***********************************

EC092     TDSPLFSSLMKGYNAVLRGSITGAQSTVNMINALWYSDGKSEAGTLKAKGSRIELGDGKH     958
M90T      TDSPLFSSLMKGYNAVLRGSITGAQSTVNMINALWYSDGKSEAGALKAKGSRIELGDGKH     960
          ********************************************:***************

EC092     FATLQVKELSADNTTFLMHTNNSWADQLNVTDKLSGSNNSVLVDFLNKPASEMSVTLITA     1018
M90T      FATLQVKELSADNTTFLMHTNNSRADQLNVTDKLSGSNNSVLVDFLNKPASEMSVTLITA     1020
          *********************** ************************************

EC092     PKGSDEKTFTAGTQQIGFSNVTPVISTEKTDDATKWVLTGYQTTADAGASKAAKDFMASG     1078
M90T      PKGSDEKTFTAGTQQIGFSNVTPVISTEKTDDATKWVLTGYQTTADAGASKAAKDFMASG     1080
          ************************************************************

EC092     YKSFLTEVNNLNKRMGDLRDTQGDAGVWARIMNGTGSADGDYSDNYTHVQIGVDRKHELD     1138
M90T      YKSFLTEVNNLNKRMGDLRDTQGDAGVWARIMNGTGSADGDYSDNYTHVQIGVDRKHELD     1140
          ************************************************************

EC092     GVDLFTGALLTYTDSNASSHAFSGKTKSVGGGLYASALFNSGAYFDLIGKYLHHDNQHTA     1198
M90T      GVDLFTGALLTYTDSNASSHAFSGKNKSVGGGLYASALFNSGAYFDLIGKYLHHDNQHTA     1200
          *************************.**********************************
```

```
EC092      NFASLGTKDYSSHSWYAGAEVGYRYHLTKESWVEPQIELVYGSVSGKAFSWEDRGMALSM     1258
M90T       NFASLGTKDYSSHSWYAGAEVGYRYHLTKESWVEPQIELVYGSVSGKAFSWEARGMALSM     1260
           **************************************************** *******

EC092      KDKDYNPLIGRTGVDVGRAFSGDDWKITARAGLGYQFDLLANGETVLQDASGEKRFEGEK     1318
M90T       KDKDYNPLIGRTGVDVGRAFSGDDWKITARAGLGYQFDLLANGETVLQDASGEKRFEGEK     1320
           ************************************************************

EC092      DSRMLMTVGMNAEIKDNMRLGLELEKSAFGKYNVDNAINANFRYVF   1364
M90T       DSRMLMTVGMNAEIKDNMRLGLELEKSAFGKYNVDNAINANFRYVF   1366
           **********************************************
```

Complete alignment between the amino acid sequence of the SepA protein of the EC092 strain and the prototype strain *Shigella flexneri* M90T (GenBank accession number: CP037923.1). Alignment was performed on the virtual platform of Cluster Omega and 21 amino acid changes were identified (orange color). The intact serine protease motif (GDSGS) was located in both strains (yellow color). The catalytic triad (His134, Asp162 and Ser268) is marked in green and the conserved site of the linker domain in light blue.
