# Supplementary material for: Genomic Dissection of an Enteroaggregative Escherichia coli Strain Isolated from Bacteremia Reveals Insights into Its Hybrid Pathogenic Potential
Source: Int J Mol Sci. 2024 Aug 26;25(17):9238. doi: 10.3390/ijms25179238 (PMC11394720; doi:10.3390/ijms25179238)
Supplement: Supplementary file 1 [file ijms-25-09238-s001.zip › Fig. S5.pdf]

Fig. S5. Alignment between the predicted amino acid sequences of the AggR protein of strains EC092 and EAEC 042.

```
042         MKLKQNIEKEIIKINNIRIHQYTVLYTSNCTIDVYTKEGSNTYLRHELIFLERGINISVR     60
EC092       MKLKQNIEKEIIKINNIRIHQYTVLYTSNCTIDVYTKEGSNTYLRHELIFLERGINISVR     60
            ************************************************************

042         LQKKKSTAKPFIAIRLNSDTLRRLKDALMIIYGISKVDACSCPNWSKGIIVADADDSVLD     120
EC092       LQKKKSTAKPFIAIRLNSDTLRRLKDALMIIYGISKVDACSCPNWSKGIIVADADDSVLD     120
            ************************************************************

042         TFKSIEHNDDSRIASDLIYLISKIENNRKIIESIYISAVSFFSDKVRNTIEKDLSKRWTL     180
EC092       TFKSIEHNDDSRIASDLIYLISKIENNRKIIESIYISAVSFFSDKVRNTIEKDLSKRWTL     180
            ************************************************************

042         AIIADEFNVSEITIRKRLESERITFNQILMQSRMSKAALLLLDNSYQISQISNMIGFSST     240
EC092       AIIADEFNVSEITIRKRLESERITFNQILMQSRMSKAALLLLDNSYQISQISNMIGFSST     240
            ************************************************************

042         SYFIRLFVKHFGVTPKQFLTYFKSQ     265
EC092       SYFIRLFVKHFGVTPKQFLTYFKSQ     265
            *************************
```

Complete alignment between the amino acid sequence of the AggR protein of the EC092 strain and the prototype strain EAEC042 (GenBank accession number: WP_011666414.1). The alignment was performed on the Cluster Omega virtual platform and showed 100% identity between the sequences.
