# Supplementary material for: Genomic Dissection of an Enteroaggregative Escherichia coli Strain Isolated from Bacteremia Reveals Insights into Its Hybrid Pathogenic Potential
Source: Int J Mol Sci. 2024 Aug 26;25(17):9238. doi: 10.3390/ijms25179238 (PMC11394720; doi:10.3390/ijms25179238)
Supplement: Supplementary file 1 [file ijms-25-09238-s001.zip › Fig. S6.pdf]

Fig. S6. Alignment between the predicted amino acid sequences of the AaiC protein from strains EC092 and EAEC 042.

```
042         MVGVMSNSSALSREFVKIEIGGKVIKGNSKVENSGDYFDGHCPQAFHAFSGIDGTVFDLI     60
EC092       MVGVMSNSSALSREFVKIEIGGKVIKGNSKVENSGDYFDGHCPQAFHAFSGIDGTVFDLI     60
            ************************************************************

042         NLKLRVTKHIQDLLENFFKRGEKEVKIEILRRESTKSGSAYSSFKVVYQGCRMHNMLLAH     120
EC092       NLKLRVTKHIQDLLENFFKRGEKEVKIEILRRESTKSGSAYSSFKVVYQGCRMHNMLLAH     120
            ************************************************************

042         DNDSNLILELSFTPEESVSIEMNIPSDDGKSTEKLGPMTYNLYKEALV 168
EC092       DNDSNLILELSFTPEESVSIEMNIPSDDGKSTEKLGPMTYNLYKEALV 168
            ************************************************
```

Complete alignment between the amino acid sequence of the AaiC protein of the EC092 strain and the prototype strain EAEC 042 (GenBank accession number: WP_000233190.1). The alignment was performed on the Cluster Omega virtual platform and showed 100% identity between the sequences.
