# Supplementary material for: Genomic Dissection of an Enteroaggregative Escherichia coli Strain Isolated from Bacteremia Reveals Insights into Its Hybrid Pathogenic Potential
Source: Int J Mol Sci. 2024 Aug 26;25(17):9238. doi: 10.3390/ijms25179238 (PMC11394720; doi:10.3390/ijms25179238)
Supplement: Supplementary file 1 [file ijms-25-09238-s001.zip › Fig. S7.pdf]

Fig. S7. Alignment between the predicted amino acid sequences of the AatA protein of strains EC092 and EAEC 042.

```
042          MRRILYSFLALNAYLFSTQTLAKDCIIDNFFQKSIQFNSYSLDIEELDINKHNNIKTMLP      60
EC092        MRRILYSFLALNAYLFSTQTLAKDCIIDNFFQKSIQFNSYSLDIEELDINKHNNIKTMLP      60
             ************************************************************

042          DINIGLGQYINNNQWFSSITDSNFYLSLSYNLLSAYEAKMQNDKLDIANYLKYIEMLSER      120
EC092        DINIGLGQYINNNQWFSSITDSNFYLSLSYNLLSAYEAKMQNDKLDIANYLKYIEMLSER      120
             ************************************************************

042          NSYIINLFSEIINYKIKKSHLMLMHERYRKFDKEYKIAKRKMSIGLISVLDVEMRYNILQ      180
EC092        NSYIINLFSEIINYKIKKSHLMLMHERYRKFDKEYKIAKRKMSIGLISVLDVEMRYNILQ      180
             ************************************************************

042          KIRFDIDALEEEERLLSDKISREYHVPESAIQDITYHKLKECKTADFYTLLAENNRLKIK      240
EC092        KIRFDIDALEEEERLLSDKISREYHVPESAIQDITYHKLKECKTADFYTLLAENNRLKIK      240
             ************************************************************

042          AADIDNDIRKLSEIPSFYLSFGLTPKQGGALGNMSLRKMDYSASLGISFPLMGLFVSSEN      300
EC092        AADIDNDIRKLSEIPSFYLSFGLTPKQGGALGNMSLRKMDYSASLGISFPLMGLFVSSEN      300
             ************************************************************

042          QKEKIISMSRARNESLKENIKLDLLEKEIRQKVDRLEKNLAMMTNELTLKRRKVEYINYR      360
EC092        QKEKIISMSRARNESLKENIKLDLLEKEIRQKVDRLEKNLAMMTNELTLKRRKVEYINYR      360
             ************************************************************

042          VKNGQDDVINYLSSVEDLHETENEFQKIGYEIEYYSLYHYFLLQHLSNKGGM      412
EC092        VKNGQDDVINYLSSVEDLHETENEFQKIGYEIEYYSLYHYFLLQHLSNKGGM      412
             ****************************************************
```

Complete alignment between the amino acid sequence of the AatA protein of the EC092 strain and the prototype strain EAEC 042 (GenBank accession number: WP_011666433.1). The alignment was performed on the Cluster Omega virtual platform and showed 100% identity between the sequences.
