# Supplementary material for: Genomic Dissection of an Enteroaggregative Escherichia coli Strain Isolated from Bacteremia Reveals Insights into Its Hybrid Pathogenic Potential
Source: Int J Mol Sci. 2024 Aug 26;25(17):9238. doi: 10.3390/ijms25179238 (PMC11394720; doi:10.3390/ijms25179238)
Supplement: Supplementary file 1 [file ijms-25-09238-s001.zip › Fig. S8.pdf]

Fig. S8. Alignment between the predicted amino acid sequences of the Aap protein of strains EC092 and EAEC 042.

```
042         MKKIKFVIFSGILGISLNAFAGGSGWSADNVDPSQCIKLSGVQYTYNNGASVCMQGFNEG     60
EC092       MKKIKFVIFSGILGISLNAFAGGSGWSADNVDPSQCIKLSGVQYTYNNGASVCMQGFNEG     60
            ************************************************************

042         KVRGVSVSGVFYYNDGTTSNFKGVVTPSTPVNTNQDIKKTNKVGVQKYRALTEWVK 116
EC092       KVRGVSVSGVFYYNDGTTSNFKGVVTPSTPVNTNQDIKKTNKVGVQKYRALTEWVK 116
            ********************************************************
```

Complete alignment between the amino acid sequence of the Aap protein from strain EC092 and the prototype strain EAEC 042 (GenBank accession number: WP_011666513.1). The alignment was performed on the Cluster Omega virtual platform and showed 100% identity between the sequences.
