# Supplementary material for: Genomic Dissection of an Enteroaggregative Escherichia coli Strain Isolated from Bacteremia Reveals Insights into Its Hybrid Pathogenic Potential
Source: Int J Mol Sci. 2024 Aug 26;25(17):9238. doi: 10.3390/ijms25179238 (PMC11394720; doi:10.3390/ijms25179238)
Supplement: Supplementary file 1 [file ijms-25-09238-s001.zip › Table S1.pdf]

Table S1. Sequencing and cleaning statistics

RAW			PostFastp			FastP step reduction			
**Number of Reads - RAW**	**Number of bases - RAW**	**Coverage - RAW**	**Number of Reads - PostFastp**	**Number of bases – PostFastp**	**Coverage - PostFastp**	**Reduction of reads**	**Base reduction**	**Reads kept**	**Bases maintained**
27.616.022	6.931.621.522	1213,6	25.595.943	5.192.852.717	1012,0	7,3%	25,1%	92,7%	74,9%

A total of 27,616,022 genomic DNA fragments (reads) were sequenced, comprising 6,931,621,522 sequenced bases. From this total, adapter sequences, low quality bases, small reads and unmatched reads were removed, resulting in 25,595,943 reads and 5,192,852,717 bases, a reduction of approximately 7.3% and 25.1% in the total sequenced respectively. This represents a coverage of 1,012 times, considering an average genome size of *E. coli* of 5,131,220 or 973 times considering the EC092 draft genome size.
