# Supplementary material for: Genomic Dissection of an Enteroaggregative Escherichia coli Strain Isolated from Bacteremia Reveals Insights into Its Hybrid Pathogenic Potential
Source: Int J Mol Sci. 2024 Aug 26;25(17):9238. doi: 10.3390/ijms25179238 (PMC11394720; doi:10.3390/ijms25179238)
Supplement: Supplementary file 1 [file ijms-25-09238-s001.zip › Table S2.pdf]

Table S2. EC092 genome assembly summary statistics

Feature	Value
# *contigs* (>= 0 bp)	527
# *contigs* (>= 200 bp)	473
# *contigs* (>= 500 bp)	195
# *contigs* (>= 1000 bp)	134
# *contigs* (>= 5000 bp)	86
# *contigs* (>= 10000 bp)	63
# *contigs* (>= 25000 bp)	51
# *contigs* (>= 50000 bp)	31
Major *contig*	581.579
Genome length (>= 0 bp)	5,344.302
Genome length (>= 200 bp)	5,335.514
Genome length (>= 500 bp)	5,241.430
Genome length (>= 1000 bp)	5,202.778
Genome length (>= 5000 bp)	5,080.286
Genome length (>= 10000 bp)	4,920.934
Genome length (>= 25000 bp)	4,748.153
Genome length (>= 50000 bp)	4.076.145
N50	121.302
N75	52.100
L50	12
L75	29
GC (%)	50.62
# N's	300
# N's for 100 kb	5.72

The size of the annotated genome contig was 5.336.090 bp (considering only contigs >=200 bases). In addition, N50 and L50 metrics were used to evaluate the quality of the assembly. The N50 of the EC092 genome assembly resulted in 121,302 bases, which corresponds to the size of the contig closest to the accumulated 50% size of the genome, when all contigs are ordered by size, from biggest to smaller. The L50 measure was 12 contigs and, similarly, corresponds to the number of contigs in the accumulated 50% size of the genome, ordered from biggest to smaller. Thus, the higher the N50 values and the lower the L50 values, the lower the fragmentation of the assembled genome and the higher its contiguity. All these statistics were calculated using the assembly quality software QUAST (GUREVICH et al., 2013).
