# Supplementary material for: Genomic Dissection of an Enteroaggregative Escherichia coli Strain Isolated from Bacteremia Reveals Insights into Its Hybrid Pathogenic Potential
Source: Int J Mol Sci. 2024 Aug 26;25(17):9238. doi: 10.3390/ijms25179238 (PMC11394720; doi:10.3390/ijms25179238)
Supplement: Supplementary file 1 [file ijms-25-09238-s001.zip › Table S3.pdf]

Table S3. Antimicrobial resistance genes identified in EC092 by ResFinder.

**Antimicrobial group**	**Gene**
β-lactamases	*(Bla)Penicillin_Binding_Protein_Ecoli*
	(Bla)AmpC1_Ecoli
	(Bla)AmpC2_Ecoli
	(Bla)ampH_Ecoli
	(Bla)blaTEM-150
	blaTEM-1A_1
Tetraciclines	*(Tet)tetR*
	(Tet)tetA
Sulfonamides	*(Sul)sul2*
	(Tmt)dfrA8_1
Aminoglycosides	*aph (3'')-Ib_5*
	aph (6)-Id_1
