# Supplementary material for: Genomic Dissection of an Enteroaggregative Escherichia coli Strain Isolated from Bacteremia Reveals Insights into Its Hybrid Pathogenic Potential
Source: Int J Mol Sci. 2024 Aug 26;25(17):9238. doi: 10.3390/ijms25179238 (PMC11394720; doi:10.3390/ijms25179238)
Supplement: Supplementary file 1 [file ijms-25-09238-s001.zip › Table S4.pdf]

Table S4. Sequence identity and amino acid changes in SPATE proteins present in EC092

SPATE	Identity[a]	No. of amino acid exchanges	Catalytic triad[b]	Serine protease motif (GDSGS)	Reference
**Pet**	99.46%	7	His124, Asp153, Ser260	Conserved	GCA_000027125.1
**Pic**	99.78%	3	His127, Asp155, Ser258	Conserved	GCA_000027125.1
**Sat**	99.38%	8	His121, Asp149, Ser256	Conserved	AAG30168.1
**SepA**	98,39%	20	His134, Asp162, Ser268	Conserved	CP037923.1

[a] The sequence identity values represent the percentage of identical amino acids in the SPATE proteins of EC092 compared to their respective reference sequences: Pet and Pic from EAEC 042, Sat from UPEC CFT073 and SepA from *Shigella flexneri* M90T.

[b] The catalytic triad consists of three key amino acids: histidine (His), aspartate (Asp), and serine (Ser). These residues, which position is described for each protein, are essential for the proteolytic activity of serine proteases, facilitating the hydrolysis of peptide bonds. The presence of a conserved serine protease motif (GDSGS) further indicates the functional integrity of these enzymes.
